# A computational study on the structure–function relationships of plant caleosins

**DOI:** 10.1038/s41598-022-26936-y

**Published:** 2023-01-02

**Authors:** Fatemeh Saadat

**Affiliations:** Biotechnology Department, College of Agriculture and Natural Resources, University of Tehran, Tehran, 4111, Iran

**Keywords:** Computational biology and bioinformatics, Plant sciences

## Abstract

Plant cells store energy in oil bodies constructed by structural proteins such as oleosins and caleosins. Although oil bodies usually accumulate in the seed and pollen of plants, caleosins are present in various organs and organelles. This issue, coupled with the diverse activities of caleosins, complicates the description of these oleo-proteins. Therefore, the current article proposes a new classification based on the bioinformatics analysis of the transmembrane topology of caleosins. Accordingly, the non-membrane class are the most abundant and diverse caleosins, especially in lower plants. Comparing the results with other reports suggests a stress response capacity for these caleosins. However, other classes play a more specific role in germination and pollination. A phylogenetic study also revealed two main clades that were significantly different in terms of caleosin type, expression profile, molecular weight, and isoelectric point (*P* < 0.01). In addition to the biochemical significance of the findings, predicting the structure of caleosins is necessary for constructing oil bodies used in the food and pharmaceutical industries.

## Introduction

Caleosins are known as the structural proteins in the nano-oil body construction^1^. The first caleosin was discovered in response to osmotic stress in rice seeds in 1996^2^. The naming is due to the presence of a calcium-binding motif involved in the Ca^2+^-signalling pathway in response to biotic and abiotic stresses^3,4^. Compared to oleosins, which are mostly found in seed or pollen oil bodies of the higher plants, caleosins exist in microsomes and almost all plant tissues, microalgae, and fungi^3^. Therefore, it is conceivable that caleosins play a role beyond the construction of oil bodies.

The various activities of caleosins raise the possibility of having different molecular structures. The best-known model for oleosin and caleosin has been described according to the hydropathy analysis, in which a central non-cytoplasmic (N) domain is flanked by two cytoplasmic (C) domains (hereafter, this structure is briefly called CNC)^5^. A proline knot motif in the central domain is responsible for mooring into the oil body. Thus, the terminal domains are supported to settle on the phospholipid layer in contact with the cytoplasm. However, our investigations show that CNC is not the only functional arrangement of caleosins. Though several bioinformatics analyses have been conducted on oleosins and caleosins^6–11^, to our knowledge, this is the first time that the presence of transmembrane (TM) domains has been included in the description and classification of caleosins.

## Results

### Classification of caleosins

**Figure 1:**
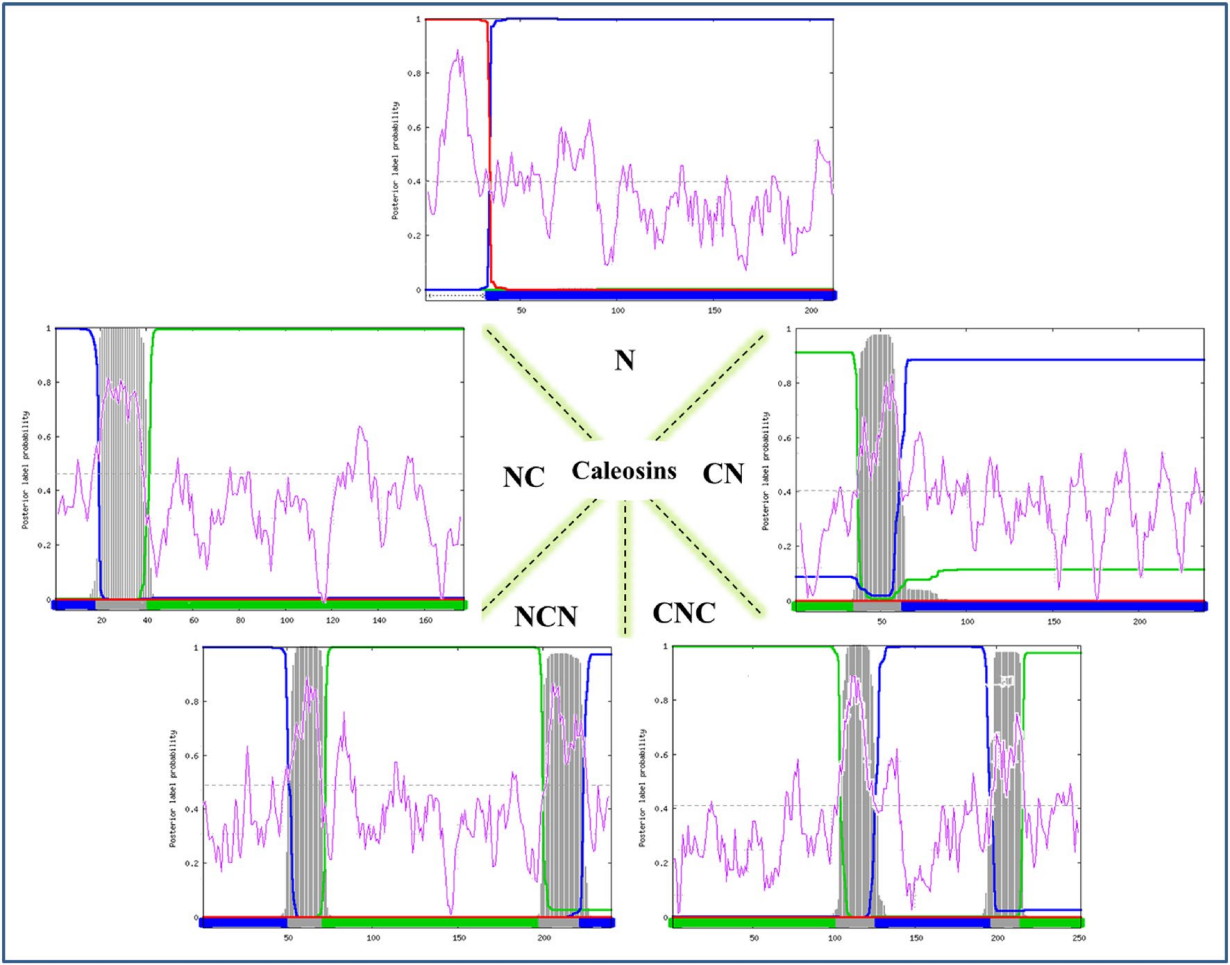
Caleosin classification according to the transmembrane topology by Phobius. The signal peptide, cytoplasmic and non-cytoplasmic regions are indicated with red, green and blue lines, respectively. The purple line shows the hydrophobic analysis by ProtScale. The zero score is drawn with a black dashed line.

**Figure 2:**
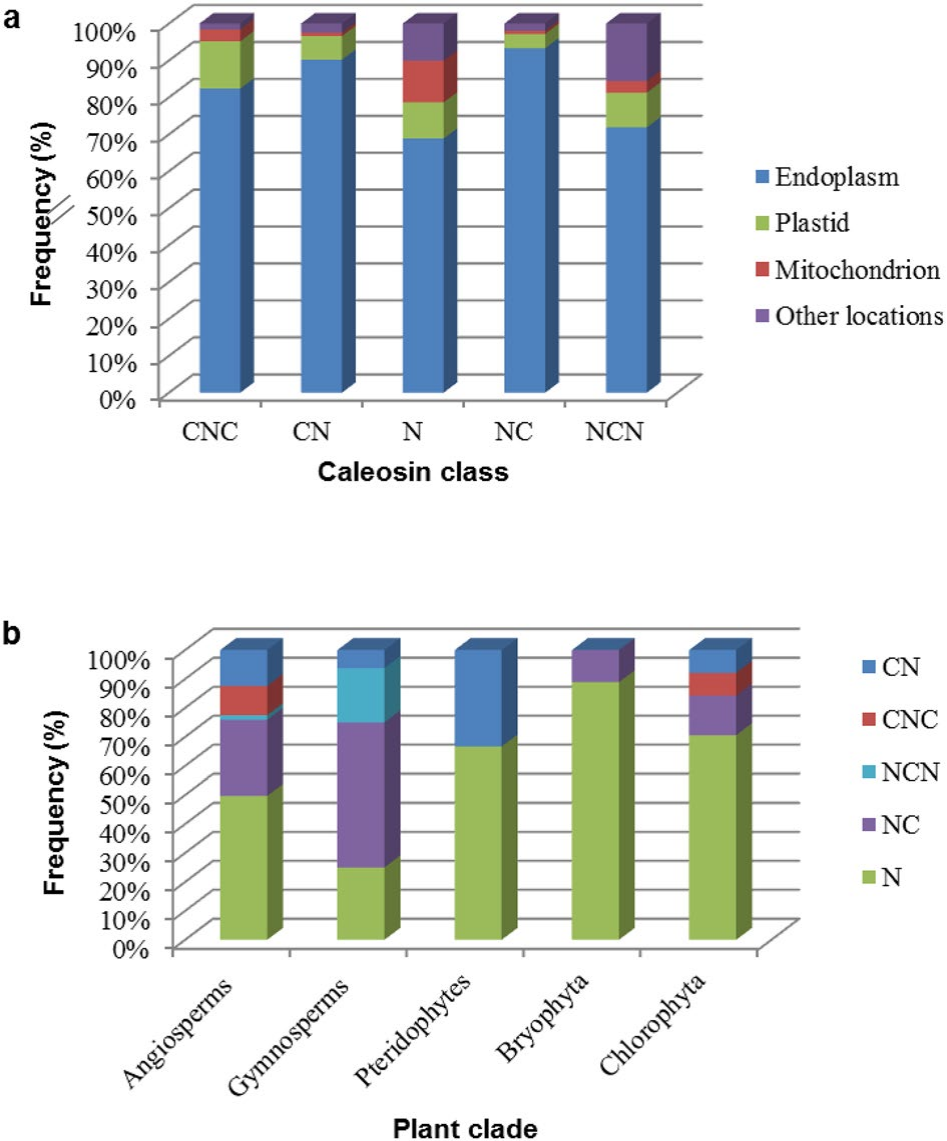
(**a**) The frequency of subcellular locations for each caleosin class. (**b**) The percentage of each caleosin class in the major clades of plants. The caleosin classes have been named based on the order of non-cytoplasmic (N) and cytoplasmic (C) domains.

A total of 1877 plant caleosins were extracted from UniProt (supplementary file). Then, they were classified into five classes according to the transmembrane topology. Each group was named based on the domain arrangement. An overview of the classification is shown in Fig. 1. Briefly, most caleosins (51% sequences) possess no transmembrane domain and are classified as non-cytoplasmic proteins (N-class). By contrast, the well-known CNC structure accounts for 10% of caleosins. In the other two classes, sequences are without the cytoplasmic domain in either N-terminus (NC-class) or C-terminus (CN-class). Finally, 2% of caleosins remains for NCN structure in which the cytoplasmic region is flanked by the non-cytoplasmic domains. The criteria and characteristics of each class are summarized in Table 1. Notably, a signal peptide was detected in some of the sequences with a non-cytoplasmic N-terminus (Table 1). A computational study by DeepLoc 1.0 predicted the endoplasmic reticulum as the predominant subcellular location of caleosins. Then, the plastid and mitochondrion were assigned to the caleosins (Fig. 2a).

**Table 1:** Classification of caleosins based on the number of the transmembrane domain (TM) and domain arrangement.

Class	Initial domain	TM counts	SP	Frequency (%)
N	Non-cytoplasmic	0	Yes (23%)	51
NC	Non-cytoplasmic	1*	Yes (5%)	25
CN	Cytoplasmic	1*	No	12
NCN	Non-cytoplasmic	2**	Yes (15%)	2
CNC	Cytoplasmic	2**	No	10

The frequency of the classes and signal peptide (SP) is also presented.

*TM might have an odd number of repeats.

**TM might have an even number of repeats.

The mentioned frequency in Table 1 mainly represents angiosperms since they include 95% of the collected data. However, the N-class is the most abundant caleosin in almost all plant clades, as displayed in Fig. 2b. According to the statistical analysis, the molecular weight of caleosins is higher in Chlorophyta than in angiosperms (*P* < 0.01) and gymnosperms (*P* < 0.05).

The motif study revealed four regions (Table 2), all of which belonged to the caleosin-related protein family (PF05042). The motif arrangement and distance were almost conserved in all classes (*P* < 0.01). However, N- and NC-classes showed more substitutions in the sequences.

**Table 2:**
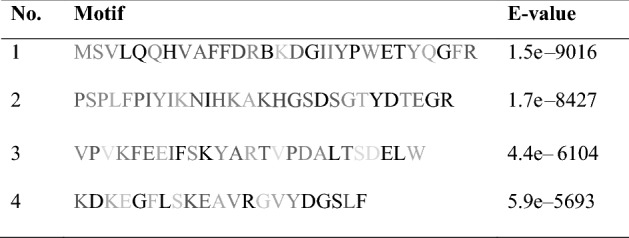
Caleosin motifs discovered using MEME web server.

The more conserved a residue, the darker the letter.

### Phylogenetic analysis

A phylogenetic tree was generated by the MAFFT algorithm used for minimum linkage analysis. Accordingly, two main clades were created in agreement with the previous report^12^. In general, clade I included ~ 90% of the CNCs and CNs, while ~ 70% of the NC caleosins, as well as half of the N and NCN members, were placed in clade II. Relatively similar clades (~ 85%) were obtained using the sequences of motifs 3 and 4.

More investigations revealed no significant difference in the acetylation of the two clades. However, average MW and pI values were higher in clades I and II, respectively (*P* < 0.01). Formerly, caleosins have been divided into H- and L- isoforms according to MW, in which L-isoform was suggested to evolve from H-isoform^7^.

### Expression profiles of caleosin genes

**Figure 3:**
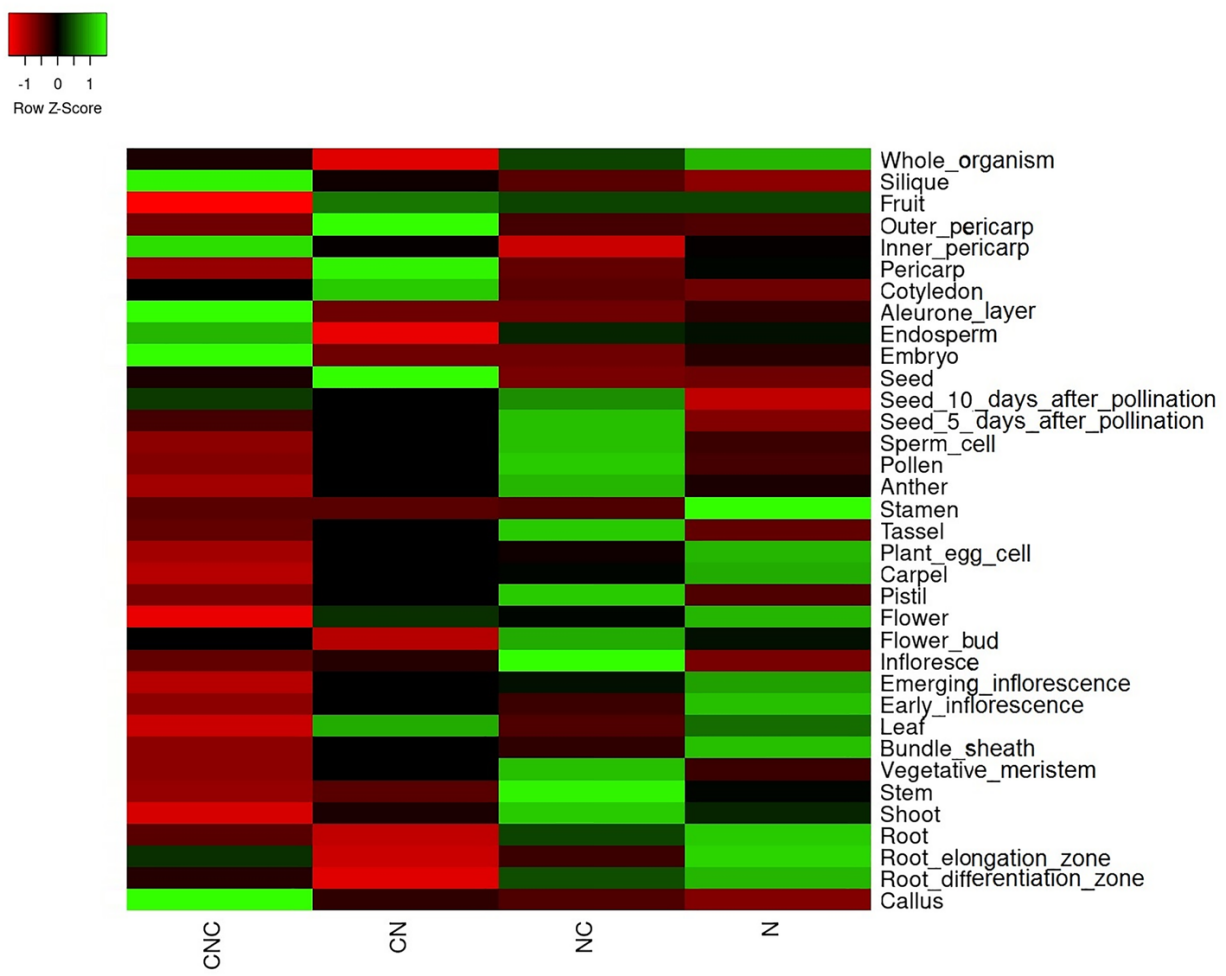
The average expression of the caleosin classes in each tissue. The caleosin classes have been named based on the order of non-cytoplasmic (N) and cytoplasmic (C) domains.

The expression profiles of 154 caleosin genes from 17 plant species were extracted from the Expression Atlas database. The average expression of each class is summarized as a heatmap in Fig. 3.

Although a combination of caleosins is expressed all over the plant, CNCs, CNs, and NCs are accumulated more specifically in parts of seed, fruit and flower, respectively. By contrast, Ns are ubiquitous, whose high expression have been reported in flower, fruit, seed, leaf, and root. The highest average expression level belonged to some CNCs predominantly found in the embryo and the aleurone layer.

**Figure 4:**
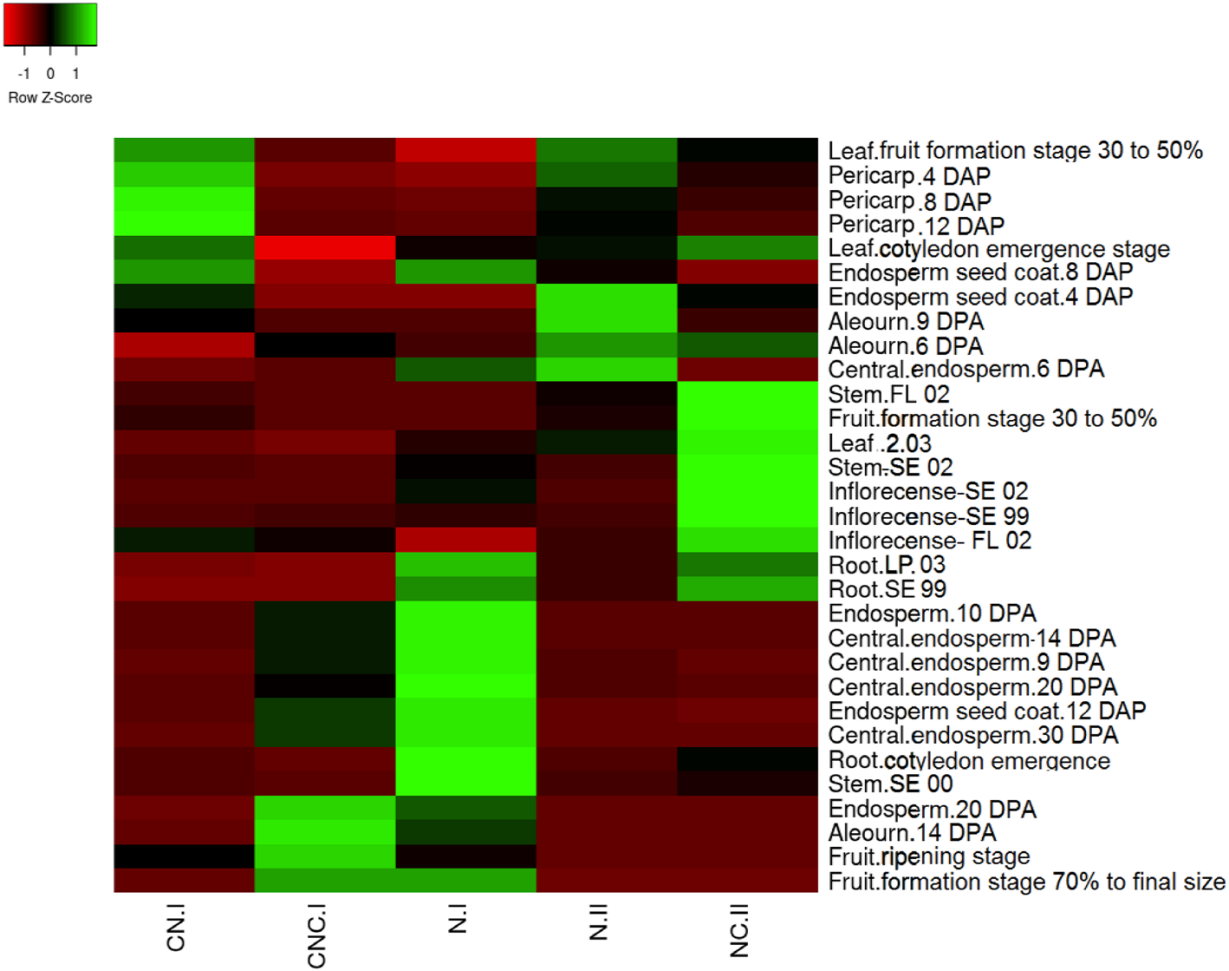
Expression profile of wheat caleosins through different developmental stages^13^. The caleosin classes have been named based on the order of non-cytoplasmic (N) and cytoplasmic (C) domains. Moreover, the phylogenetic clade (I or II) is mentioned next to the name of each caleosin class. DPA: days post-anthesis; DAP: days after pollination.

The RNA-seq of wheat aleurone and starchy layers has revealed a decrease in NCs and a considerable increase in CNCs during the post-anthesis^13^. However, the results were diverse for Ns, which could be related to their phylogenetic clade (Fig. 4).

Comparing the expression profile with the phylogenetic tree suggested that high-expressed embryonic caleosins (e.g. Q7XQ03 and C5YBZ6) might belong to clade I, while floral caleosins (e.g. C5Z7Z3 and C5XZA3) could be located in clade II. However, statistical verification requires more data, especially about the remaining CNCs and NCs in their non-dominant clade.

### Abiotic and biotic stress response

**Figure 5:**
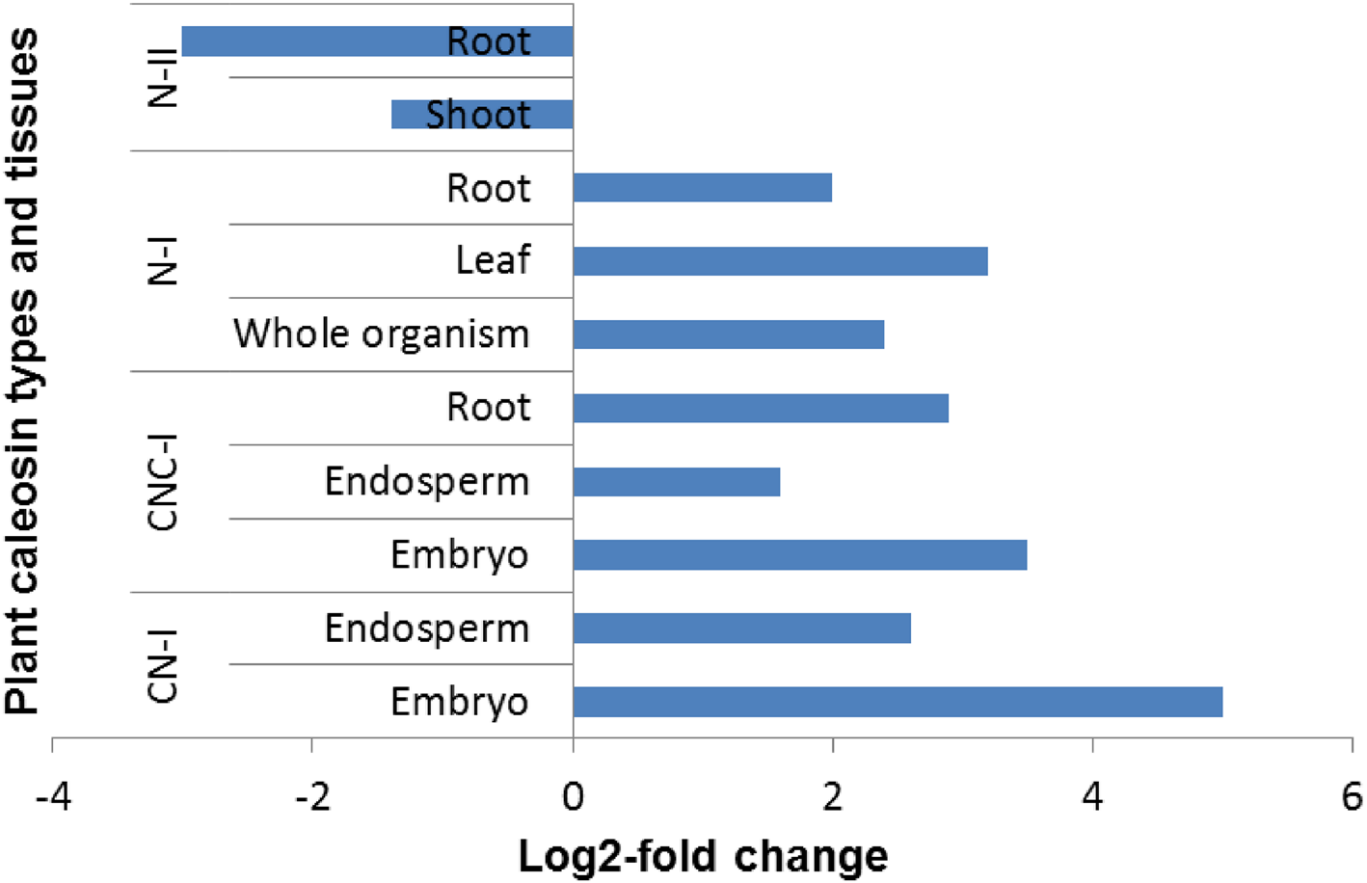
The differential expression of caleosins in response to 20 Mm abscisic acid. The data was extracted from the Expression Atlas database. The caleosin classes have been named based on the order of non-cytoplasmic (N) and cytoplasmic (C) domains. Moreover, the phylogenetic clade (I or II) is mentioned next to the name of each caleosin class.

Abscisic acid (ABA) and osmotic stress upregulate caleosins and promote seed dormancy^14^. However, expression patterns differ depending on the tissue, developmental stage, type of caleosin and some unknown factors. An investigation of the differential expression shows the upregulation of clade I caleosins in response to ABA (Fig. 5). Moreover, there is a significant difference (*P* < 0.05) between N-II and caleosins of clade I in response to drought. A notable example of clade I is RD20, expressed predominantly in non-seed tissues, and regulates stomatal closures in response to drought^15^. By contrast, D6PW68, as an N-II caleosin, is upregulated more by salicylic acid (SA) and methyl jasmonate (MeJA) than ABA^16^. The same results have been reported for some N-II caleosins from cotton^17^ and rice^18^. Interestingly, each class of *GhCLO* genes have shown almost similar expression patterns; for example, the NC-II caleosins (*GhCLO1*, *GhCLO10 *and *GhCLO11*) in response to NaCl, PEG and MeJA, and the CN-I caleosins (*GhCLO16* and *GhCLO7*) in response to ABA^17^.

Although there were no reports on NC caleosins in the Expression Atlas database, AtCLO4 and AtCLO7, as NC-II caleosins, have been reported to downregulate in response to ABA and salt stress^19,20^. By contrast, A2XVG1 and Q7FAX1 (NC-I) are upregulated in response to ABA^2^, PEG6000 and drought^18,21^.

Further investigation of expression profiles revealed no significant differences between caleosins in response to biotic stress. RD20 is a well-studied caleosin strongly induced by the reactive oxygen species (ROS) caused by pathogens. Some reports indicated interaction of RD20 with an α-dioxygenase in leaf lipid droplets catalyzes the conversion of α-linolenic acid to the antifungal phytoalexin^22,23^.

### Comparison of topology prediction approaches

Membrane proteins are underrepresented in the PDB database (∼ 1–2% of all available structures) due to their difficulty in crystallization. Therefore, alternative experimental and computational methods are considered for membrane proteins. Here we compare the Phobius results with other algorithms and research to make a more accurate assumption.

The possibility of a transmembrane structure was strengthened by the description of caleosin as an insoluble and non-cytoplasmic protein in 1996^2^. Furthermore, the role of the hydrophobic central region of membrane proteins of oil bodies has been confirmed by structural domain deletion^24^, protease protection assay^25^, and structural proteomic approach^26^. All the mentioned methods suggest a CNC structure for an oleosin from *Arabidopsis* *thaliana* (P29525), in agreement with computational predictions. However, not all algorithms always make identical outcomes, and not all predictions will be supported by experiments.

**Figure 6:**
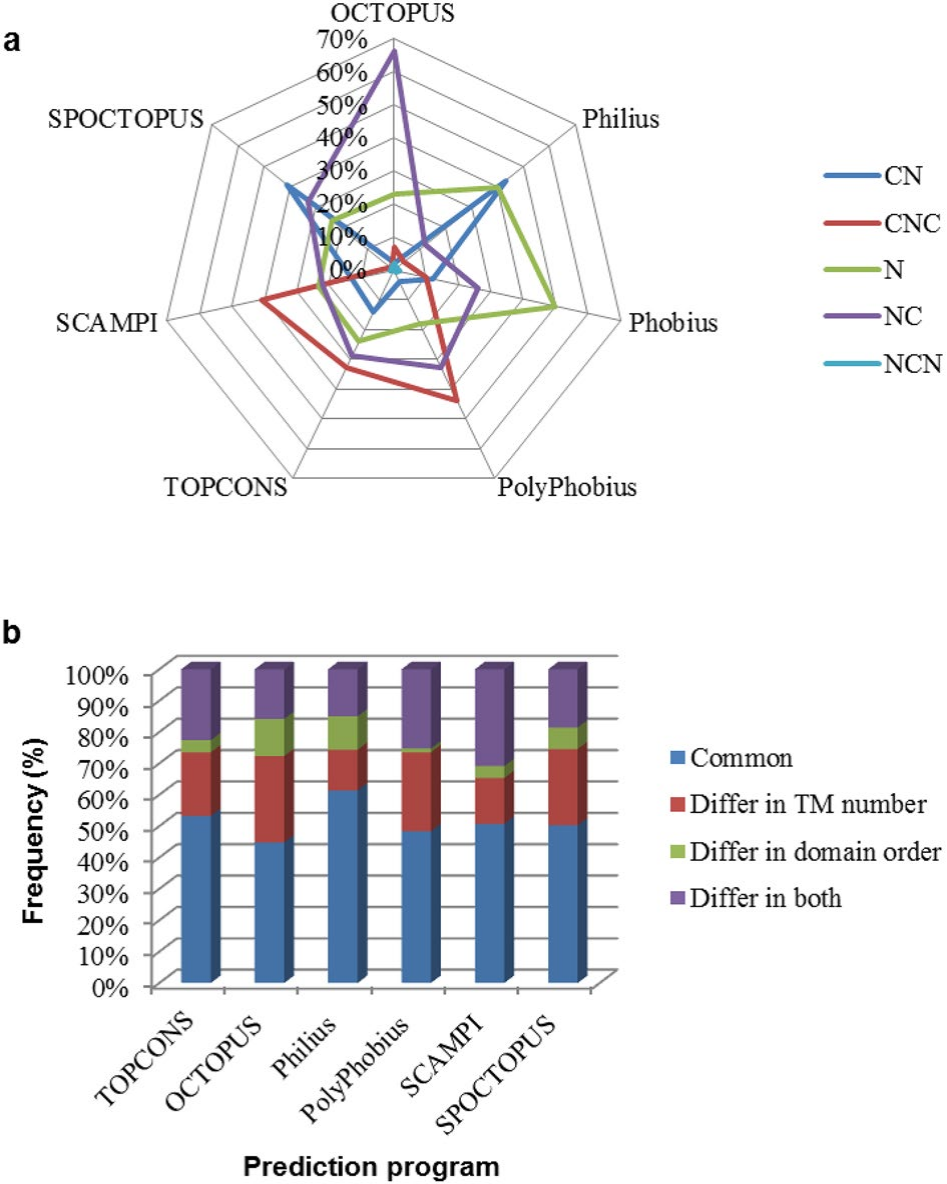
(**a**) Transmembrane topology of caleosins detected by Philius, Phobius, PolyPhobius, OCTOPUS, SCAMPI, SPOCTOPUS, and TOPCONS. (**b**) The frequency of the same results between Phobius and other algorithms and the diversities resulting from the number of TM, domain order or both.

As illustrated in Fig. 6a, most caleosins belonged to N-class by Philius and Phobius, while the tendency was more towards CNC-class by TOPCONS, SCAMPI and PolyPhobius. However, the percentage of NC- and NCN-classes was almost constant. Comparing the predictions showed that nearly half of the results were the same as Phobius, and most differences were related to the number of TMs (Fig. 6b). The highest overlap occurred between TOPCONS and SCAMPI with 87% similarity, while OCTOPUS had less than 50% nearness to other algorithms (supplementary data).

More investigations indicated that TOPCONS and Phobius make a better distinction between the phylogenetic clades. These programs also have shown the highest performance in topology prediction of transmembrane and globular proteins containing signal peptides^27^.

Comparing the predictions with the published enzymatic digestion experiments showed that Phobius is more consistent with laboratory data. For example, the microsome-associated AtCLO3 is protected from proteinase K digestion^3^. It is more in line with the Phobius prediction, which proposed an N structure. However, the secondary structure of caleosins is dramatically affected by the polarity of environments^28^. Regardless of how close the predictions are to the experiments, the results can be considered a suitable descriptor for distinguishing the location and function of caleosins.

## Discussion

After the first description of caleosins in the 1990s, many studies focused on their structures and functions. Unlike oleosins, caleosins are ubiquitous proteins with a wide range of activities, from the response to stress to seed development. Although CNC has been known as the main structure of the oleo-proteins, caleosins have been recently divided into three groups based on the hydrophobic domain position that could be in the centre, N-terminus or random sites (e.g., algae and fungal caleosins)^29^. However, the importance of the transmembrane topology was overlooked. Consequently, in this article, five classes were introduced (Table 1) that could explain the functions and locations of plant caleosins.

Reviewing other research met our results and partly opened up the knot of this puzzle. Accordingly, most CN and CNC caleosins had been reported as seed-specific proteins^6,9,30–44^, while NC members had been isolated from the floral parts^9,17,42,45–51^. That might explain the higher portion of NC and CNC in gymnosperms and angiosperms, respectively (Fig. 2).

Furthermore, the N members are expressed in almost all plant tissues, including root^52^, flower^42,53^, seed^21,37,54^, and leaf^9,16,55^. Hydrophobicity is expected to localize N caleosins outside the cells to sense the exogenous stimuli and participate in the signal transition. However, optical coherence tomography revealed them on the periphery of the plasma membrane, passed through the epicyte and extended into the cytoplasm^16^. It has been reported that Ns plays a role in basal resistance to drought^18,44^, salinity^29^, low temperature^56,57^, and pathogen infection^16^, as well as reaction with various substrates in the oxylipin pathway^58,59^. The best-known member of the N-class, RD20 or AtCLO3, is upregulated following exposure to stress, which leads to the production of oxidized fatty acids in the ABA and salicylic acid signalling pathways. Lipid oxidation reduces ROS levels, minor cell death, and delayed floral transition^60,61^. Additionally, the *rd20* knock-out plants have enhanced stomatal opening and reduced tolerance to drought^62^.

Although N-class is the prevalent caleosin in plants and bacteria, a combination of classes exists in fungi with the superiority of CNCs (~ 40%) (data not shown). Besides, CNC- and NC-classes account for 90% of plant oleosins and are mainly expressed in seed and flower parts, respectively (data not shown). It is in agreement with other structural classifications of oleosins^8,11^. This data resolves the importance and role of each class of oleo-proteins.

## Methods

### Characteristics of caleosin proteins

Protein sequences were collected using “caleosin” as a keyword in UniProt version 2022–11 (http://uniprot.org/). The results were limited to viridiplantae in the taxonomy section. Then, a domain exploration was done for the caleosins in Phobius (http://phobius.sbc.su.se)^63^ and TOPCONS (https://topcons.cbr.su.se)^27^. Subsequently, the hydropathic plot was produced by ProtScale (http://web.expasy.org/protscale/) using the Kyte-Doolittle method^64^. Furthermore, the subcellular location, acetylation, and theoretical isoelectric point (pI) and molecular weight (MW) values were predicted by DeepLoc - 1.0 (https://services.healthtech.dtu.dk/service.php?DeepLoc-1.0)^65^, NetAcet 1.0 Server (http://www.cbs.dtu.dk/services/NetAcet/)^66^, and Expasy (https://web.expasy.org/compute_pi/)^67^, respectively.

### Motif analysis of the caleosins

The dataset was analyzed using MEME Version 5.2.0 (meme-suite.org/tools/meme)^68^ with zero or one occurrence per sequence for four different motifs. The annotations of the motifs were investigated using the GenomeNet website (http://www.genome.jp/tools/motif/).

### Gene expression profile analysis

The gene expression level of plant caleosins was obtained from Expression Atlas database (https://www.ebi.ac.uk/gxa/home). The data were plotted by Heatmapper with default parameters and Pearson distance measurement (www.heatmapper.ca/)^69^.

### Protein alignment and phylogenetic study

Multiple alignments and phylogenetic analyses were constructed by MAFFT (https://mafft.cbrc.jp/alignment/server/index.html) with default parameters^70^.

### Statistical analysis

Data were subjected to analysis of variance (ANOVA), t-test and Wilcoxon test using R software (R-3.6.3). The P values less than 0.05 were taken into consideration.

## Supplementary Information

Supplementary Information.

## Data Availability

The datasets generated during and/or analysed during the current study are available from the corresponding author on reasonable request.

## Abbreviations

ABA: Abscisic acid
C: Cytoplasmic domain
DAP: Days after pollination
DPA: Days post-anthesis
H-isoform: High molecular weight isoform
L-isoform: Low molecular weight isoform
MeJA: Methyl jasmonate
MW: Molecular weight
N: Non-cytoplasmic domain
ROS: Reactive oxygen species
pI: Isoelectric point
SA: Salicylic acid
SP: Signal peptide
TM: Transmembrane region

## References

[1] Saadat F, Macheroux P, Alizadeh H, Razavi SH (2022). Economic purification of recombinant uricase by artificial oil bodies. Bioresour. Bioprocess..

[2] Frandsen G, Müller-Uri F, Nielsen M, Mundy J, Skriver K (1996). Novel plant Ca-binding protein expressed in response to abscisic acid and osmotic stress. J. Biol. Chem..

[3] Partridge M, Murphy DJ (2009). Roles of a membrane-bound caleosin and putative peroxygenase in biotic and abiotic stress responses in *Arabidopsis*. Plant Physiol. Biochem..

[4] Shimada TL, Hara-Nishimura I (2015). Leaf oil bodies are subcellular factories producing antifungal oxylipins. Curr. Opin. Plant Biol..

[5] Purkrtová Z, Chardot T, Froissard M (2015). N-terminus of seed caleosins is essential for lipid droplet sorting but not for lipid accumulation. Arch. Biochem. Biophys..

[6] Hyun TK, Kumar D, Cho YY, Hyun HN, Kim JS (2013). Computational identification and phylogenetic analysis of the oil-body structural proteins, oleosin and caleosin, in castor bean and flax. Gene.

[7] Shen Y (2014). Genomic analysis and expression investigation of caleosin gene family in *Arabidopsis*. Biochem. Biophys. Res. Commun..

[8] Huang M-D, Huang AHC (2015). Bioinformatics reveal five lineages of oleosins and the mechanism of lineage evolution related to structure/function from green algae to seed plants. Plant Physiol..

[9] Shen Y (2016). Identification, duplication, evolution and expression analyses of caleosins in *Brassica* plants and *Arabidopsis* subspecies. Mol. Genet. Genom..

[10] Rahman F (2018). Evolutionary and genomic analysis of the caleosin/peroxygenase (CLO/PXG) gene/protein families in the *Viridiplantae*. PLoS ONE.

[11] Yuan Y (2021). Genome-wide identification and analysis of *Oleosin* gene family in four cotton species and its involvement in oil accumulation and germination. BMC Plant Biol..

[12] Hanano A (2006). Plant seed peroxygenase is an original heme-oxygenase with an EF-hand calcium binding motif. J. Biol. Chem..

[13] Pingault L (2015). Deep transcriptome sequencing provides new insights into the structural and functional organization of the wheat genome. Genome Biol..

[14] van der Schoot C, Paul LK, Paul SB, Rinne PLH (2011). Plant lipid bodies and cell-cell signaling. Plant Signal. Behav..

[15] Sharma R, Vishal P, Kaul S, Dhar MK (2017). Epiallelic changes in known stress-responsive genes under extreme drought conditions in *Brassica juncea* (L.) Czern. Plant Cell Rep..

[16] Feng H (2011). Cloning and characterization of a calcium binding EF-hand protein gene TaCab1 from wheat and its expression in response to *Puccinia striiformis* f. sp. and abiotic stresses. Mol. Biol. Rep..

[17] Fu X (2022). Evolution and stress responses of CLO genes and potential function of the GhCLO06 gene in salt resistance of cotton. Front. Plant Sci..

[18] Wei Z, Ma H, Ge X (2011). Phylogenetic analysis and drought-responsive expression of the rice caleosin gene family. Sci. Bull..

[19] Kim YY, Jung KW, Yoo KS, Jeung JU, Shin JS (2011). A stress-responsive caleosin-like protein, AtCLO4, acts as a negative regulator of ABA responses in *Arabidopsis*. Plant Cell Physiol..

[20] Brunetti SC, Arseneault MKM, Gulick PJ (2022). The caleosin CLO7 and its role in the heterotrimeric G-protein signalling network. J. Plant Physiol..

[21] Jing P (2021). OsClo5 functions as a transcriptional co-repressor by interacting with OsDi19-5 to negatively affect salt stress tolerance in rice seedlings. Plant J..

[22] Brocard L (2017). Proteomic analysis of lipid droplets from *Arabidopsis* aging leaves brings new insight into their biogenesis and functions. Front. Plant Sci..

[23] Shimada TL, Takano Y, Hara-Nishimura I (2015). Oil body-mediated defense against fungi: From tissues to ecology. Plant Signal. Behav..

[24] van Rooijen GJH, Moloney MM (1995). Structural requirements of oleosin domains for subcellular targeting to the oil body. Plant Physiol..

[25] Abell BM (1997). Role of the proline knot motif in oleosin endoplasmic reticulum topology and oil body targeting. Plant Cell.

[26] Jolivet P (2017). Structural proteomics: Topology and relative accessibility of plant lipid droplet associated proteins. J. Proteom..

[27] Tsirigos KD, Peters C, Shu N, Käll L, Elofsson A (2015). The TOPCONS web server for consensus prediction of membrane protein topology and signal peptides. Nucleic Acids Res..

[28] Purkrtova Z (2007). Structural properties of caleosin: A MS and CD study. Arch. Biochem. Biophys..

[29] Charuchinda P (2015). Caleosin from *Chlorella vulgaris* TISTR 8580 is salt-induced and heme-containing protein. Biosci. Biotechnol. Biochem..

[30] Chen DH, Chyan CL, Jiang PL, Chen CS, Tzen JTC (2012). The same oleosin isoforms are present in oil bodies of rice embryo and aleurone layer while caleosin exists only in those of the embryo. Plant Physiol. Biochem..

[31] Du C (2019). Proteomic identification of lipid-bodies-associated proteins in maize seeds. Acta Physiol. Plant..

[32] Lamberti C (2020). Identification of a caleosin associated with hazelnut (*Corylus avellana* L.) oil bodies. Plant Biol..

[33] Lang S, Liu X, Ma G, Lan Q, Wang X (2014). Identification of desiccation tolerance transcripts potentially involved in rape (*Brassica napus* L.) seeds development and germination. Plant Physiol. Biochem..

[34] Liu H (2005). Characterisation and functional analysis of two barley caleosins expressed during barley caryopsis development. Planta.

[35] Liu X (2022). Multiple caleosins have overlapping functions in oil accumulation and embryo development. J. Exp. Bot..

[36] Lizong H, Shufen L, Wujun G (2013). Expression, divergence and evolution of the caleosin gene family in *Brassica rapa*. Arch. Biol. Sci..

[37] Lu HC, Jiang PL, Hsu LRC, Chyan CL, Tzen JTC (2010). Characterization of oil bodies in adlay (*Coix lachryma-jobi* L). Biosci. Biotechnol. Biochem..

[38] Meesapyodsuk D, Qiu X (2011). A peroxygenase pathway involved in the biosynthesis of epoxy fatty acids in oat. Plant Physiol..

[39] Poxleitner M, Rogers SW, Lacey Samuels A, Browse J, Rogers JC (2006). A role for caleosin in degradation of oil-body storage lipid during seed germination. Plant J. Cell Mol. Biol..

[40] Tnani H, López I, Jouenne T, Vicient CM (2011). Protein composition analysis of oil bodies from maize embryos during germination. J. Plant Physiol..

[41] Toorop PE, Barroco RM, Engler G, Groot SPC, Hilhorst HWM (2005). Differentially expressed genes associated with dormancy or germination of *Arabidopsis*
*thaliana* seeds. Planta.

[42] Umate P (2012). Comparative genomics of the lipid-body-membrane proteins oleosin, caleosin and steroleosin in magnoliophyte, lycophyte and bryophyte. Genom. Proteom. Bioinform..

[43] Vali K (2018). Characterization of an Arabidopsis thaliana Anther-Specific Caleosin.

[44] Vermachova M (2011). New protein isoforms identified within *Arabidopsis*
*thaliana* seed oil bodies combining chymotrypsin/trypsin digestion and peptide fragmentation analysis. Proteomics.

[45] Jiang PL, Chen JCF, Chiu ST, Tzen JTC (2009). Stable oil bodies sheltered by a unique caleosin in cycad megagametophytes. Plant Physiol. Biochem..

[46] Jiang PL, Jauh GY, Wang CS, Tzen JTC (2008). A unique caleosin in oil bodies of lily pollen. Plant Cell Physiol..

[47] Lu J-Y (2020). MS1, a direct target of MS188, regulates the expression of key sporophytic pollen coat protein genes in *Arabidopsis*. J. Exp. Bot..

[48] Pasaribu B, Chen CS, Liao YK, Jiang PL, Tzen JTC (2017). Identification of caleosin and oleosin in oil bodies of pine pollen. Plant Physiol. Biochem..

[49] Pasaribu B (2014). Identification of caleosin and two oleosin isoforms in oil bodies of pine megagametophytes. Plant Physiol. Biochem..

[50] Shen Y (2016). Genome-wide characterization and phylogenetic and expression analyses of the caleosin gene family in soybean, common bean and barrel medic. Arch. Biol. Sci..

[51] Zhao W, Liu J, Qian L, Guan M, Guan C (2022). Genome-wide identification and characterization of oil-body-membrane proteins in polyploid crop *Brassica napus*. Plants.

[52] Hanano A, Shaban M, Almousally I, Murphy DJ (2018). Identification of a dioxin-responsive oxylipin signature in roots of date palm: involvement of a 9-hydroperoxide fatty acid reductase, caleosin/peroxygenase PdPXG2. Sci. Rep..

[53] Wu X (2015). Proteome profiling of maize pollen coats reveals novel protein components. Plant Mol. Biol. Report..

[54] Liu H, Wang C, Chen F, Shen S (2015). Proteomic analysis of oil bodies in mature *Jatropha curcas* seeds with different lipid content. J. Proteomics.

[55] Brunetti SC (2021). The stress induced caleosin, RD20/CLO3, acts as a negative regulator of GPA1 in *Arabidopsis*. Plant Mol. Biol..

[56] Zeng X (2022). Rice OsClo5, a caleosin protein, negatively regulates cold tolerance through the jasmonate signalling pathway. Plant Biol..

[57] Khalil HB (2011). Heterotrimeric Gα subunit from wheat (*Triticum aestivum*), GA3, interacts with the calcium-binding protein, Clo3, and the phosphoinositide-specific phospholipase C, PI-PLC1. Plant Mol. Biol..

[58] Blée E, Flenet M, Boachon B, Fauconnier ML (2012). A non-canonical caleosin from *Arabidopsis* efficiently epoxidizes physiological unsaturated fatty acids with complete stereoselectivity. FEBS J..

[59] Hanano A (2017). Specific caleosin/peroxygenase and lipoxygenase activities are tissue-differentially expressed in date palm (*Phoenix dactylifera* L.) seedlings and are further induced following exposure to the toxin 2,3,7,8-tetrachlorodibenzo-p-dioxin. Front. Plant Sci..

[60] Hanano A, Bessoule J-J, Heitz T, Blée E (2015). Involvement of the caleosin/peroxygenase RD20 in the control of cell death during *Arabidopsis* responses to pathogens. Plant Signal. Behav..

[61] Blée E (2014). The reductase activity of the *Arabidopsis* caleosin responsive to dessication20 mediates gibberellin-dependent flowering time, abscisic acid sensitivity, and tolerance to oxidative stress. Plant Physiol..

[62] Aubert Y (2010). RD20, a stress-inducible caleosin, participates in stomatal control, transpiration and drought tolerance in *Arabidopsis*
*thaliana*. Plant Cell Physiol..

[63] Kall L, Krogh A, Sonnhammer ELL (2007). Advantages of combined transmembrane topology and signal peptide prediction–the Phobius web server. Nucleic Acids Res..

[64] Kyte J, Doolittle RF (1982). A simple method for displaying the hydropathic character of a protein. J. Mol. Biol..

[65] Almagro Armenteros JJ, Sønderby CK, Sønderby SK, Nielsen H, Winther O (2017). DeepLoc: Prediction of protein subcellular localization using deep learning. Bioinformatics.

[66] Kiemer L, Bendtsen JD, Blom N (2005). NetAcet: Prediction of N-terminal acetylation sites. Bioinform. Oxf. Engl..

[67] Bjellqvist B, Basse B, Olsen E, Celis JE (1994). Reference points for comparisons of two-dimensional maps of proteins from different human cell types defined in a pH scale where isoelectric points correlate with polypeptide compositions. Electrophoresis.

[68] Bailey TL, Williams N, Misleh C, Li WW (2006). MEME: Discovering and analyzing DNA and protein sequence motifs. Nucleic Acids Res..

[69] Babicki S (2016). Heatmapper: Web-enabled heat mapping for all. Nucleic Acids Res..

[70] Katoh K, Rozewicki J, Yamada KD (2017). MAFFT online service: Multiple sequence alignment, interactive sequence choice and visualization. Br. Bioinform..

