# Supplementary material for: A computational study on the structure–function relationships of plant caleosins
Source: Sci Rep. 2023 Jan 2;13:72. doi: 10.1038/s41598-022-26936-y (PMC9807586; doi:10.1038/s41598-022-26936-y)
Supplement: Supplementary file 1 — Supplementary Information. [file 41598_2022_26936_MOESM1_ESM.pdf]

**A Computational Study on the Structure-Function Relationships of Plant Caleosins**

Fatemeh Saadat

Supplementary file- The predicted transmembrane topology for caleosins.

| ID | Phobius | TOPCONS | OCTOPUS | Philius | PolyPhobius | SCAMPI | SPOCTOPUS |
|---|---|---|---|---|---|---|---|
| A0A834SQH1 | CN | CN | CN | CN | CN | CN | CN |
| A0A6L2KBG7 | CN | CN | NC | CN | CN | CN | CN |
| A0A453CIR3 | CN | CN | NC | CN | CN | CN | CN |
| A0A5N6LV89 | CN | CN | NC | CN | CN | CN | CN |
| F4KBH6 | CN | CN | NC | CN | CN | CN | CN |
| A0A453CF76 | CN | CN | NC | CN | CN | CN | CN |
| A0A453CIR7 | CN | CN | NC | CN | CN | CN | CN |
| M4CFD9 | CN | CN | NC | CN | CN | CN | CN |
| A0A0D2RYK9 | CN | CN | NC | CN | CN | CN | CN |
| A0A0D3EB99 | CN | CN | NC | CN | CN | CN | CN |
| A0A371EA81 | CN | CN | NC | CN | CN | CN | CN |
| A0A699S8K4 | CN | CN | NC | CN | CN | CN | CN |
| A0A6A4QYJ0 | CN | CN | NC | CN | CN | CN | CN |
| A0A5D2P2B3 | CN | CN | NC | CN | CN | CN | CN |
| A0A5J5UEE4 | CN | CN | NC | CN | CN | CN | CN |
| A0A5J5Q033 | CN | CN | NC | CN | CN | CN | CN |
| M8AIP2 | N | CN | NC | CN | CN | CN | CN |
| A0A453IMG5 | N | CN | NC | CN | CN | CN | CN |
| A0A1D1Y7E6 | N | CN | NC | CN | CN | CN | CN |
| A0A0Q3GRA1 | NC | CN | NC | CN | CN | CN | CN |
| A0A833QEP6 | NC | CN | NC | CN | CN | CN | CN |
| A0A392NLD2 | NC | CN | NC | CN | CN | CN | CN |
| A0A822XLI1 | NC | CN | NC | CN | CN | CN | CN |
| A0A6S7LMM6 | NC | CN | NC | CN | CN | CN | CN |
| A0A453CF66 | NC | CN | NC | CN | CN | CN | CN |
| A0A5J5U2T5 | N | CN | NC | N | CN | CN | CN |
| K4ACH7 | N | CN | NC | N | CN | CN | CN |
| C6T9L2 | CN | CN | CN | CN | CNC | CN | CN |
| A0A5N6N9U4 | CN | CN | CN | CN | CNC | CN | CN |
| A0A1Y1IP03 | CN | CN | CN | CN | CNC | CN | CN |
| A0A7I8J5S3 | N | CN | CN | CN | CNC | CN | CN |
| A0A834GHN1 | NCN | CN | CN | CN | CNC | CN | CN |
| A0A1D6JTL1 | CN | CN | NC | CN | CNC | CN | CN |
| A0A078H5N9 | CN | CN | NC | CN | CNC | CN | CN |
| A0A0D3AX43 | CN | CN | NC | CN | CNC | CN | CN |
| A0A2G5CYB5 | CN | CN | NC | CN | CNC | CN | CN |
| A0A2J6JRN6 | CN | CN | NC | CN | CNC | CN | CN |
| A0A2U1PZH9 | CN | CN | NC | CN | CNC | CN | CN |
| A0A398AFG7 | CN | CN | NC | CN | CNC | CN | CN |
| A0A3N6SA83 | CN | CN | NC | CN | CNC | CN | CN |
| A0A3P6ATU8 | CN | CN | NC | CN | CNC | CN | CN |

| | | | | | | | |
|---|---|---|---|---|---|---|---|
| A0A6J0JRN1 | CN | CN | NC | CN | CNC | CN | CN |
| A0A6J1ENI1 | CN | CN | NC | CN | CNC | CN | CN |
| A0A6J1KB76 | CN | CN | NC | CN | CNC | CN | CN |
| A0A6S7PAI9 | CN | CN | NC | CN | CNC | CN | CN |
| A0A7J0G5E8 | CN | CN | NC | CN | CNC | CN | CN |
| A0A816KLK9 | CN | CN | NC | CN | CNC | CN | CN |
| M4DVS9 | CN | CN | NC | CN | CNC | CN | CN |
| A0A151RJJ8 | CN | CN | NC | CN | CNC | CN | CN |
| A0A161YAN7 | CN | CN | NC | CN | CNC | CN | CN |
| A0A1Q3CRY5 | CN | CN | NC | CN | CNC | CN | CN |
| A0A2P6R5I3 | CN | CN | NC | CN | CNC | CN | CN |
| A0A314ZPZ4 | CN | CN | NC | CN | CNC | CN | CN |
| A0A498K2U8 | CN | CN | NC | CN | CNC | CN | CN |
| A0A4S4E3M0 | CN | CN | NC | CN | CNC | CN | CN |
| A0A540KKK8 | CN | CN | NC | CN | CNC | CN | CN |
| A0A565CP03 | CN | CN | NC | CN | CNC | CN | CN |
| A0A5E4EB46 | CN | CN | NC | CN | CNC | CN | CN |
| A0A5N5H0F6 | CN | CN | NC | CN | CNC | CN | CN |
| A0A6A5N7E1 | CN | CN | NC | CN | CNC | CN | CN |
| A0A6D2IH78 | CN | CN | NC | CN | CNC | CN | CN |
| A0A6J5VC05 | CN | CN | NC | CN | CNC | CN | CN |
| A0A6P5SBD4 | CN | CN | NC | CN | CNC | CN | CN |
| A0A7G2FFS0 | CN | CN | NC | CN | CNC | CN | CN |
| A0A7N0T8A4 | CN | CN | NC | CN | CNC | CN | CN |
| G1JSL4 | CN | CN | NC | CN | CNC | CN | CN |
| M5WJM8 | CN | CN | NC | CN | CNC | CN | CN |
| M8BZW5 | CN | CN | NC | CN | CNC | CN | CN |
| V7BE66 | CN | CN | NC | CN | CNC | CN | CN |
| V7BRK4 | CN | CN | NC | CN | CNC | CN | CN |
| A0A0D3C3W8 | CN | CN | NC | CN | CNC | CN | CN |
| A0A2K3N0P0 | CN | CN | NC | CN | CNC | CN | CN |
| A0A397ZMN1 | CN | CN | NC | CN | CNC | CN | CN |
| A0A650ATJ7 | CN | CN | NC | CN | CNC | CN | CN |
| A0A6J1D8E4 | CN | CN | NC | CN | CNC | CN | CN |
| A0A816JVZ8 | CN | CN | NC | CN | CNC | CN | CN |
| C3S7H2 | CN | CN | NC | CN | CNC | CN | CN |
| C3S7H3 | CN | CN | NC | CN | CNC | CN | CN |
| M4DZA0 | CN | CN | NC | CN | CNC | CN | CN |
| A0A251UPD6 | CN | CN | NC | CN | CNC | CN | CN |
| A0A2G5CYA6 | CN | CN | NC | CN | CNC | CN | CN |
| A0A2U1N6W3 | CN | CN | NC | CN | CNC | CN | CN |
| A0A8F5CMJ0 | CN | CN | NC | CN | CNC | CN | CN |
| C6T9L0 | CN | CN | NC | CN | CNC | CN | CN |
| D8R120 | CN | CN | NC | CN | CNC | CN | CN |
| D8S185 | CN | CN | NC | CN | CNC | CN | CN |
| Q9SQ57 | CN | CN | NC | CN | CNC | CN | CN |
| Q9FLN9 | CN | CN | NC | CN | CNC | CN | CN |

| | | | | | | | |
|---|---|---|---|---|---|---|---|
| A0A072VX57 | CN | CN | NC | CN | CNC | CN | CN |
| A0A087FWV5 | CN | CN | NC | CN | CNC | CN | CN |
| A0A0A0LHL6 | CN | CN | NC | CN | CNC | CN | CN |
| A0A0D3DC91 | CN | CN | NC | CN | CNC | CN | CN |
| A0A0R0HVK7 | CN | CN | NC | CN | CNC | CN | CN |
| A0A1J3G5B2 | CN | CN | NC | CN | CNC | CN | CN |
| A0A1J3IK81 | CN | CN | NC | CN | CNC | CN | CN |
| A0A1J3JMK5 | CN | CN | NC | CN | CNC | CN | CN |
| A0A1S2XUP0 | CN | CN | NC | CN | CNC | CN | CN |
| A0A1S3B507 | CN | CN | NC | CN | CNC | CN | CN |
| A0A3P6F6E2 | CN | CN | NC | CN | CNC | CN | CN |
| A0A5A7TFK8 | CN | CN | NC | CN | CNC | CN | CN |
| A0A5D3DZQ6 | CN | CN | NC | CN | CNC | CN | CN |
| A0A6S7LCK6 | CN | CN | NC | CN | CNC | CN | CN |
| A0A816MPL4 | CN | CN | NC | CN | CNC | CN | CN |
| D7M708 | CN | CN | NC | CN | CNC | CN | CN |
| I3SK18 | CN | CN | NC | CN | CNC | CN | CN |
| V4MLK1 | CN | CN | NC | CN | CNC | CN | CN |
| A0A8I6XFH1 | CN | CN | NC | CN | CNC | CN | CN |
| Q6UFY6 | CN | CN | NC | CN | CNC | CN | CN |
| A0A4U6TR31 | CNC | CN | NC | CN | CNC | CN | CN |
| A0A835BMC9 | CNC | CN | NC | CN | CNC | CN | CN |
| K3Y8Z6 | CNC | CN | NC | CN | CNC | CN | CN |
| A0A1R3IPG5 | CNC | CN | NC | CN | CNC | CN | CN |
| O81270 | CNC | CN | NC | CN | CNC | CN | CN |
| A0A6C0PBB1 | CNC | CN | NC | CN | CNC | CN | CN |
| A0A6P5YSG2 | CNC | CN | NC | CN | CNC | CN | CN |
| A0A453CEV5 | CNC | CN | NC | CN | CNC | CN | CN |
| A0A2K1IHR1 | N | CN | NC | CN | CNC | CN | CN |
| A0A6A6MJX7 | N | CN | NC | CN | CNC | CN | CN |
| A0A811MNZ7 | N | CN | NC | CN | CNC | CN | CN |
| V4LMZ0 | N | CN | NC | CN | CNC | CN | CN |
| A0A075C587 | N | CN | NC | CN | CNC | CN | CN |
| A0A0C9S875 | N | CN | NC | CN | CNC | CN | CN |
| A0A176W5X3 | N | CN | NC | CN | CNC | CN | CN |
| A0A2H5PWA9 | N | CN | NC | CN | CNC | CN | CN |
| A0A2U1P496 | N | CN | NC | CN | CNC | CN | CN |
| A0A498IKJ3 | N | CN | NC | CN | CNC | CN | CN |
| A0A540LEH0 | N | CN | NC | CN | CNC | CN | CN |
| A0A540NBH3 | N | CN | NC | CN | CNC | CN | CN |
| A0A540NGD2 | N | CN | NC | CN | CNC | CN | CN |
| A0A5E4EKE0 | N | CN | NC | CN | CNC | CN | CN |
| A0A7J8PDL8 | N | CN | NC | CN | CNC | CN | CN |
| A9P1Z1 | N | CN | NC | CN | CNC | CN | CN |
| A0A087GMF0 | N | CN | NC | CN | CNC | CN | CN |
| A0A6J0MM38 | N | CN | NC | CN | CNC | CN | CN |
| A0A6J0N157 | N | CN | NC | CN | CNC | CN | CN |

| | | | | | | | |
|---|---|---|---|---|---|---|---|
| A9LLF1 | N | CN | NC | CN | CNC | CN | CN |
| V4NYA1 | N | CN | NC | CN | CNC | CN | CN |
| A0A6P5TPM0 | N | CN | NC | CN | CNC | CN | CN |
| D8RV92 | N | CN | NC | CN | CNC | CN | CN |
| A0A1D6JA96 | N | CN | NC | CN | CNC | CN | CN |
| O22788 | N | CN | NC | CN | CNC | CN | CN |
| A0A1B5GE57 | N | CN | NC | CN | CNC | CN | CN |
| A0A6J5VX37 | N | CN | NC | CN | CNC | CN | CN |
| M8AUX2 | N | CN | NC | CN | CNC | CN | CN |
| R7W9Y5 | N | CN | NC | CN | CNC | CN | CN |
| W5B7W5 | N | CN | NC | CN | CNC | CN | CN |
| M8A9M9 | N | CN | NC | CN | CNC | CN | CN |
| A0A060LAL9 | NC | CN | NC | CN | CNC | CN | CN |
| A0A175YNQ4 | NC | CN | NC | CN | CNC | CN | CN |
| A0A1I9R3Y5 | NC | CN | NC | CN | CNC | CN | CN |
| A0A2G5CYC8 | NC | CN | NC | CN | CNC | CN | CN |
| A0A835RS49 | NC | CN | NC | CN | CNC | CN | CN |
| A0A0C9RUU0 | NC | CN | NC | CN | CNC | CN | CN |
| A0A2N9E534 | NC | CN | NC | CN | CNC | CN | CN |
| A0A3B6CA45 | NC | CN | NC | CN | CNC | CN | CN |
| A0A6P3ZB35 | NC | CN | NC | CN | CNC | CN | CN |
| A0A6P5TPI6 | NC | CN | NC | CN | CNC | CN | CN |
| A0A835JQW5 | NC | CN | NC | CN | CNC | CN | CN |
| A0A843TJP5 | NC | CN | NC | CN | CNC | CN | CN |
| Q7FAX1 | NC | CN | NC | CN | CNC | CN | CN |
| A0A0D3FXV1 | NC | CN | NC | CN | CNC | CN | CN |
| A0A0E0H328 | NC | CN | NC | CN | CNC | CN | CN |
| A0A0E0PBH2 | NC | CN | NC | CN | CNC | CN | CN |
| A0A118K2Z4 | NC | CN | NC | CN | CNC | CN | CN |
| A0A1S4E2Y4 | NC | CN | NC | CN | CNC | CN | CN |
| A0A2P5ANM6 | NC | CN | NC | CN | CNC | CN | CN |
| A0A314ZAG9 | NC | CN | NC | CN | CNC | CN | CN |
| A0A445J0P2 | NC | CN | NC | CN | CNC | CN | CN |
| A0A5N6QSE3 | NC | CN | NC | CN | CNC | CN | CN |
| A0A5N6QV11 | NC | CN | NC | CN | CNC | CN | CN |
| I1PMY8 | NC | CN | NC | CN | CNC | CN | CN |
| A0A8J4BEQ6 | NC | CN | NC | CN | CNC | CN | CN |
| A0A199W1C2 | NC | CN | NC | CN | CNC | CN | CN |
| A8B479 | NC | CN | NC | CN | CNC | CN | CN |
| I1L364 | NC | CN | NC | CN | CNC | CN | CN |
| A0A0D6R8H4 | NC | CN | NC | CN | CNC | CN | CN |
| A0A388L8B4 | NC | CN | NC | CN | CNC | CN | CN |
| A0A565CQT3 | NC | CN | NC | CN | CNC | CN | CN |
| A0A7J7P3E2 | NC | CN | NC | CN | CNC | CN | CN |
| A9P0Q5 | NC | CN | NC | CN | CNC | CN | CN |
| B8XX15 | NC | CN | NC | CN | CNC | CN | CN |
| W1PLQ7 | NC | CN | NC | CN | CNC | CN | CN |

| | | | | | | | |
|---|---|---|---|---|---|---|---|
| A0A6J5TD55 | NC | CN | NC | CN | CNC | CN | CN |
| Q94B09 | NC | CN | NC | CN | CNC | CN | CN |
| A0A453CIN7 | NC | CN | NC | CN | CNC | CN | CN |
| A0A2J7ZX97 | NC | CN | NC | CN | CNC | CN | CN |
| A0A124SEK3 | CNC | CNC | NC | CNC | CNC | CN | CN |
| A0A2P5Y7K3 | CNC | CNC | NC | CNC | CNC | CN | CN |
| A0A2G5CYB6 | CNC | CNC | NC | CNC | CNC | CN | CN |
| A0A1U8KAM7 | N | CN | NC | N | CNC | CN | CN |
| A0A5D2J870 | N | CN | NC | N | CNC | CN | CN |
| D8S1Z4 | N | CN | NC | N | CNC | CN | CN |
| A0A176W6L6 | N | CN | NC | N | CNC | CN | CN |
| A0A383VMH7 | N | CN | NC | N | CNC | CN | CN |
| A0A5C7IC74 | NCN | CN | CN | NCN | CNC | CN | CN |
| A0A453CF71 | N | CN | NC | CN | N | CN | CN |
| B3SL30 | N | CN | NC | N | N | CN | CN |
| B3SL16 | N | CN | NC | N | N | CN | CN |
| B3SL17 | N | CN | NC | N | N | CN | CN |
| B3SL18 | N | CN | NC | N | N | CN | CN |
| B3SL38 | N | CN | NC | N | N | CN | CN |
| B3SL39 | N | CN | NC | N | N | CN | CN |
| A0A022PVL1 | CN | CN | CN | CN | NC | CN | CN |
| A0A565BW00 | CN | CN | CN | CN | NC | CN | CN |
| A0A2K3KQ57 | NC | CN | CN | CN | NC | CN | CN |
| A0A0R0EQZ7 | NC | CN | CN | CN | NC | CN | CN |
| A0A453CEY0 | NC | CN | CN | CN | NC | CN | CN |
| A0A0B2S8E8 | NC | CN | NC | CN | NC | CN | CN |
| A0A1D6JTL3 | NC | CN | NC | CN | NC | CN | CN |
| A0A453CF97 | NC | CN | NC | CN | NC | CN | CN |
| A9NKI8 | NC | CN | NC | CN | NC | CN | CN |
| A0A199UZA5 | NC | CN | NC | CN | NC | CN | CN |
| A0A7J7LPC1 | NC | CN | NC | CN | NC | CN | CN |
| A9NU18 | NC | CN | NC | CN | NC | CN | CN |
| D8S798 | N | CN | NC | N | NC | CN | CN |
| D8SZ23 | N | CN | NC | N | NC | CN | CN |
| A0A2K1JPR2 | N | NCN | NC | N | NC | CN | CN |
| A0A804RKX5 | N | CN | NC | CN | NCN | CN | CN |
| A0A804LGA9 | N | CN | NC | CN | NCN | CN | CN |
| A0A2R6XAZ2 | N | CN | NC | N | NCN | CN | CN |
| A0A6S7M3C0 | CN | CN | NC | CN | CN | CNC | CN |
| A0A2K3DQ17 | N | CN | NC | N | CN | CNC | CN |
| A0A6A2Z104 | N | CN | NC | N | CN | CNC | CN |
| A0A8J4BF47 | NC | CN | NC | N | CN | CNC | CN |
| A0A0S3SPK1 | CN | CN | CN | CN | CNC | CNC | CN |
| A0A835HX17 | CNC | CN | CN | CN | CNC | CNC | CN |
| A0A2P6QRD5 | CNC | CN | CN | CN | CNC | CNC | CN |
| A0A438GYI2 | N | CN | CN | CN | CNC | CNC | CN |
| A0A7I8KWJ0 | N | CN | CN | CN | CNC | CNC | CN |

| | | | | | | | |
|---|---|---|---|---|---|---|---|
| A0A6A4L7G4 | NCN | CN | CN | CN | CNC | CNC | CN |
| A0A4D6KLU4 | CN | CNC | NC | CN | CNC | CNC | CN |
| A0A0B0MW71 | CN | CNC | NC | CN | CNC | CNC | CN |
| A0A2G9I7G6 | CN | CNC | NC | CN | CNC | CNC | CN |
| A0A2J6M3Z0 | CN | CNC | NC | CN | CNC | CNC | CN |
| A0A565C0T7 | CN | CNC | NC | CN | CNC | CNC | CN |
| A0A6P4M459 | CN | CNC | NC | CN | CNC | CNC | CN |
| A0A7C9D9I5 | CN | CNC | NC | CN | CNC | CNC | CN |
| A0A7J6VVU0 | CN | CNC | NC | CN | CNC | CNC | CN |
| A9P193 | CN | CNC | NC | CN | CNC | CNC | CN |
| I1JRR6 | CN | CNC | NC | CN | CNC | CNC | CN |
| A0A0B2RDP7 | CN | CNC | NC | CN | CNC | CNC | CN |
| A0A0L9VCU0 | CN | CNC | NC | CN | CNC | CNC | CN |
| A0A0L9VLP5 | CN | CNC | NC | CN | CNC | CNC | CN |
| A0A0S3S550 | CN | CNC | NC | CN | CNC | CNC | CN |
| A0A175YPE8 | CN | CNC | NC | CN | CNC | CNC | CN |
| A0A1S3TCL6 | CN | CNC | NC | CN | CNC | CNC | CN |
| A0A1U8FBJ6 | CN | CNC | NC | CN | CNC | CNC | CN |
| A0A251VLH6 | CN | CNC | NC | CN | CNC | CNC | CN |
| A0A2G2VJ78 | CN | CNC | NC | CN | CNC | CNC | CN |
| A0A2G3B4S5 | CN | CNC | NC | CN | CNC | CNC | CN |
| A0A2G5DPR4 | CN | CNC | NC | CN | CNC | CNC | CN |
| A0A2J6M443 | CN | CNC | NC | CN | CNC | CNC | CN |
| A0A2R6QAU3 | CN | CNC | NC | CN | CNC | CNC | CN |
| A0A3B6DFM9 | CN | CNC | NC | CN | CNC | CNC | CN |
| A0A3Q0FEI3 | CN | CNC | NC | CN | CNC | CNC | CN |
| A0A445CP56 | CN | CNC | NC | CN | CNC | CNC | CN |
| A0A453CF92 | CN | CNC | NC | CN | CNC | CNC | CN |
| A0A453CFA3 | CN | CNC | NC | CN | CNC | CNC | CN |
| A0A5J4XCV0 | CN | CNC | NC | CN | CNC | CNC | CN |
| A0A6A5LEQ4 | CN | CNC | NC | CN | CNC | CNC | CN |
| A0A6C0QI92 | CN | CNC | NC | CN | CNC | CNC | CN |
| A0A6C0QJR5 | CN | CNC | NC | CN | CNC | CNC | CN |
| A0A6P4DMY5 | CN | CNC | NC | CN | CNC | CNC | CN |
| A0A6S7LEG5 | CN | CNC | NC | CN | CNC | CNC | CN |
| A0A7J6VLM9 | CN | CNC | NC | CN | CNC | CNC | CN |
| A0A834SJ15 | CN | CNC | NC | CN | CNC | CNC | CN |
| A0A8B8KZK8 | CN | CNC | NC | CN | CNC | CNC | CN |
| A0A8B8M9E2 | CN | CNC | NC | CN | CNC | CNC | CN |
| V7BI35 | CN | CNC | NC | CN | CNC | CNC | CN |
| A0A199VWN6 | CN | CNC | NC | CN | CNC | CNC | CN |
| A0A1U8I295 | CN | CNC | NC | CN | CNC | CNC | CN |
| A0A3Q7I8T6 | CN | CNC | NC | CN | CNC | CNC | CN |
| A0A6P5EKN1 | CN | CNC | NC | CN | CNC | CNC | CN |
| A0A0D2Q517 | CN | CNC | NC | CN | CNC | CNC | CN |
| A0A1J7I2W7 | CN | CNC | NC | CN | CNC | CNC | CN |
| A0A2G5CYB7 | CN | CNC | NC | CN | CNC | CNC | CN |

| | | | | | | | |
|---|---|---|---|---|---|---|---|
| A0A2G9GPD7 | CN | CNC | NC | CN | CNC | CNC | CN |
| A0A2G9I7H9 | CN | CNC | NC | CN | CNC | CNC | CN |
| A0A2P5FAM0 | CN | CNC | NC | CN | CNC | CNC | CN |
| A0A4D8XPJ2 | CN | CNC | NC | CN | CNC | CNC | CN |
| A0A5D2JFC3 | CN | CNC | NC | CN | CNC | CNC | CN |
| A0A6P5X0P7 | CN | CNC | NC | CN | CNC | CNC | CN |
| A0A7C9D5B5 | CN | CNC | NC | CN | CNC | CNC | CN |
| A0A7C9DB10 | CN | CNC | NC | CN | CNC | CNC | CN |
| A0A803MTV2 | CN | CNC | NC | CN | CNC | CNC | CN |
| A0A6S7M5K6 | CN | CNC | NC | CN | CNC | CNC | CN |
| A0A6D2I7G1 | CN | CNC | NC | CN | CNC | CNC | CN |
| A0A6S7LEF7 | CN | CNC | NC | CN | CNC | CNC | CN |
| A0A178USE6 | CN | CNC | NC | CN | CNC | CNC | CN |
| A0A4D6N1J7 | CN | CNC | NC | CN | CNC | CNC | CN |
| A0A5S9YEF5 | CN | CNC | NC | CN | CNC | CNC | CN |
| D7MKF7 | CN | CNC | NC | CN | CNC | CNC | CN |
| A0A072U607 | CN | CNC | NC | CN | CNC | CNC | CN |
| A0A078FVX3 | CN | CNC | NC | CN | CNC | CNC | CN |
| A0A078FZZ9 | CN | CNC | NC | CN | CNC | CNC | CN |
| A0A0D2NN46 | CN | CNC | NC | CN | CNC | CNC | CN |
| A0A151T132 | CN | CNC | NC | CN | CNC | CNC | CN |
| A0A1J3DXT0 | CN | CNC | NC | CN | CNC | CNC | CN |
| A0A1J3I7D0 | CN | CNC | NC | CN | CNC | CNC | CN |
| A0A1S2XXQ4 | CN | CNC | NC | CN | CNC | CNC | CN |
| A0A2G9GPC9 | CN | CNC | NC | CN | CNC | CNC | CN |
| A0A397XQQ4 | CN | CNC | NC | CN | CNC | CNC | CN |
| A0A3P6DAN8 | CN | CNC | NC | CN | CNC | CNC | CN |
| A0A3P6E7E6 | CN | CNC | NC | CN | CNC | CNC | CN |
| A0A3Q7JD10 | CN | CNC | NC | CN | CNC | CNC | CN |
| A0A4D9APK9 | CN | CNC | NC | CN | CNC | CNC | CN |
| A0A5D2B6Z5 | CN | CNC | NC | CN | CNC | CNC | CN |
| A0A5N6M8K8 | CN | CNC | NC | CN | CNC | CNC | CN |
| A0A6I9TVW0 | CN | CNC | NC | CN | CNC | CNC | CN |
| A0A6N2B3P0 | CN | CNC | NC | CN | CNC | CNC | CN |
| A0A811M7E1 | CN | CNC | NC | CN | CNC | CNC | CN |
| A0A8J5YJP1 | CN | CNC | NC | CN | CNC | CNC | CN |
| M1CV68 | CN | CNC | NC | CN | CNC | CNC | CN |
| Q4JHI1 | CN | CNC | NC | CN | CNC | CNC | CN |
| R0FYF8 | CN | CNC | NC | CN | CNC | CNC | CN |
| V4LGR5 | CN | CNC | NC | CN | CNC | CNC | CN |
| W9SF93 | CN | CNC | NC | CN | CNC | CNC | CN |
| A0A022QXG8 | CN | CNC | NC | CN | CNC | CNC | CN |
| A0A1B5GDR6 | CN | CNC | NC | CN | CNC | CNC | CN |
| A0A1B5GEQ5 | CN | CNC | NC | CN | CNC | CNC | CN |
| A0A3B6C9M9 | CN | CNC | NC | CN | CNC | CNC | CN |
| A0A453CEZ4 | CN | CNC | NC | CN | CNC | CNC | CN |
| A0A6V7QMU5 | CN | CNC | NC | CN | CNC | CNC | CN |

| | | | | | | | |
|---|---|---|---|---|---|---|---|
| M7ZYZ8 | CN | CNC | NC | CN | CNC | CNC | CN |
| A0A2G2YAH8 | CN | CNC | NC | CN | CNC | CNC | CN |
| A0A6P5YTB8 | CN | CNC | NC | CN | CNC | CNC | CN |
| A0A7J8LZ70 | CN | CNC | NC | CN | CNC | CNC | CN |
| A0A7J8X906 | CN | CNC | NC | CN | CNC | CNC | CN |
| A0A7J8LZ33 | CN | CNC | NC | CN | CNC | CNC | CN |
| A0A7J8X8X6 | CN | CNC | NC | CN | CNC | CNC | CN |
| M8CMZ5 | CN | CNC | NC | CN | CNC | CNC | CN |
| A0A8I6X5M2 | CN | CNC | NC | CN | CNC | CNC | CN |
| A0A0B0MVG6 | CN | CNC | NC | CN | CNC | CNC | CN |
| A0A1U8HZ45 | CN | CNC | NC | CN | CNC | CNC | CN |
| A0A5D2FAW1 | CN | CNC | NC | CN | CNC | CNC | CN |
| A0A5D2XU67 | CN | CNC | NC | CN | CNC | CNC | CN |
| A0A6A3D362 | CN | CNC | NC | CN | CNC | CNC | CN |
| A0A3B6B144 | CNC | CNC | NC | CN | CNC | CNC | CN |
| A0A0D3DN16 | CNC | CNC | NC | CN | CNC | CNC | CN |
| C5WSN8 | CNC | CNC | NC | CN | CNC | CNC | CN |
| A0A061EMW3 | CNC | CNC | NC | CN | CNC | CNC | CN |
| A0A0B2R887 | CNC | CNC | NC | CN | CNC | CNC | CN |
| A0A0D2RRR7 | CNC | CNC | NC | CN | CNC | CNC | CN |
| A0A2N9I485 | CNC | CNC | NC | CN | CNC | CNC | CN |
| A0A3L6PKF7 | CNC | CNC | NC | CN | CNC | CNC | CN |
| A0A445IPS5 | CNC | CNC | NC | CN | CNC | CNC | CN |
| A0A5J9WIK2 | CNC | CNC | NC | CN | CNC | CNC | CN |
| A0A6I9R4H7 | CNC | CNC | NC | CN | CNC | CNC | CN |
| A0A6P5X2C4 | CNC | CNC | NC | CN | CNC | CNC | CN |
| A0A811MRA5 | CNC | CNC | NC | CN | CNC | CNC | CN |
| A0A811MSS8 | CNC | CNC | NC | CN | CNC | CNC | CN |
| A0A835AV07 | CNC | CNC | NC | CN | CNC | CNC | CN |
| B9SQU8 | CNC | CNC | NC | CN | CNC | CNC | CN |
| C0P5W8 | CNC | CNC | NC | CN | CNC | CNC | CN |
| O23959 | CNC | CNC | NC | CN | CNC | CNC | CN |
| A0A1E5VR32 | CNC | CNC | NC | CN | CNC | CNC | CN |
| A0A2N9G5D3 | CNC | CNC | NC | CN | CNC | CNC | CN |
| A0A3L6GAU3 | CNC | CNC | NC | CN | CNC | CNC | CN |
| A0A4Y7KUH2 | CNC | CNC | NC | CN | CNC | CNC | CN |
| A0A6A1W362 | CNC | CNC | NC | CN | CNC | CNC | CN |
| A0A6S7PJK3 | CNC | CNC | NC | CN | CNC | CNC | CN |
| A0A811QMF4 | CNC | CNC | NC | CN | CNC | CNC | CN |
| A0A811R1E1 | CNC | CNC | NC | CN | CNC | CNC | CN |
| A0A833QVY0 | CNC | CNC | NC | CN | CNC | CNC | CN |
| B4FKP4 | CNC | CNC | NC | CN | CNC | CNC | CN |
| F2DG99 | CNC | CNC | NC | CN | CNC | CNC | CN |
| A0A0D9ZN24 | CNC | CNC | NC | CN | CNC | CNC | CN |
| A0A0E0H326 | CNC | CNC | NC | CN | CNC | CNC | CN |
| A0A0E0PBH0 | CNC | CNC | NC | CN | CNC | CNC | CN |
| A0A1S4A2P1 | CNC | CNC | NC | CN | CNC | CNC | CN |

| | | | | | | | |
|---|---|---|---|---|---|---|---|
| A0A2G5CY97 | CNC | CNC | NC | CN | CNC | CNC | CN |
| A0A6N2BU85 | CNC | CNC | NC | CN | CNC | CNC | CN |
| B9FG41 | CNC | CNC | NC | CN | CNC | CNC | CN |
| I1PMY6 | CNC | CNC | NC | CN | CNC | CNC | CN |
| A0A067EG10 | CNC | CNC | NC | CN | CNC | CNC | CN |
| A0A2G9GQ74 | CNC | CNC | NC | CN | CNC | CNC | CN |
| A0A2H5P3I1 | CNC | CNC | NC | CN | CNC | CNC | CN |
| A0A5B6YPB8 | CNC | CNC | NC | CN | CNC | CNC | CN |
| A0A5C7IV57 | CNC | CNC | NC | CN | CNC | CNC | CN |
| A0A7C8YD61 | CNC | CNC | NC | CN | CNC | CNC | CN |
| M0WMG7 | CNC | CNC | NC | CN | CNC | CNC | CN |
| W5BA38 | CNC | CNC | NC | CN | CNC | CNC | CN |
| A0A0Q3P3Y0 | CNC | CNC | NC | CN | CNC | CNC | CN |
| A0A6A6MNW4 | CNC | CNC | NC | CN | CNC | CNC | CN |
| A0A059PZ24 | CNC | CNC | NC | CN | CNC | CNC | CN |
| A0A0D9W725 | CNC | CNC | NC | CN | CNC | CNC | CN |
| A0A2S3I8E1 | CNC | CNC | NC | CN | CNC | CNC | CN |
| A0A2T7CX94 | CNC | CNC | NC | CN | CNC | CNC | CN |
| A0A328EAY3 | CNC | CNC | NC | CN | CNC | CNC | CN |
| A0A4U6TAJ8 | CNC | CNC | NC | CN | CNC | CNC | CN |
| A0A5J9TV62 | CNC | CNC | NC | CN | CNC | CNC | CN |
| A0A835BN48 | CNC | CNC | NC | CN | CNC | CNC | CN |
| A0A843VBI2 | CNC | CNC | NC | CN | CNC | CNC | CN |
| J3LZI0 | CNC | CNC | NC | CN | CNC | CNC | CN |
| M7Z243 | CNC | CNC | NC | CN | CNC | CNC | CN |
| M8CJY2 | CNC | CNC | NC | CN | CNC | CNC | CN |
| W5B8D6 | CNC | CNC | NC | CN | CNC | CNC | CN |
| W5BUF4 | CNC | CNC | NC | CN | CNC | CNC | CN |
| A0A087GH35 | CNC | CNC | NC | CN | CNC | CNC | CN |
| A0A0D3A6R2 | CNC | CNC | NC | CN | CNC | CNC | CN |
| A0A1S3XAB6 | CNC | CNC | NC | CN | CNC | CNC | CN |
| A0A1S3Z6X8 | CNC | CNC | NC | CN | CNC | CNC | CN |
| A0A1U7W7T7 | CNC | CNC | NC | CN | CNC | CNC | CN |
| A0A2G9I7G7 | CNC | CNC | NC | CN | CNC | CNC | CN |
| A0A2H5P2Q6 | CNC | CNC | NC | CN | CNC | CNC | CN |
| A0A2I0IZE8 | CNC | CNC | NC | CN | CNC | CNC | CN |
| A0A314LBD2 | CNC | CNC | NC | CN | CNC | CNC | CN |
| A0A397YBJ2 | CNC | CNC | NC | CN | CNC | CNC | CN |
| A0A398AMC8 | CNC | CNC | NC | CN | CNC | CNC | CN |
| A0A3N6PR09 | CNC | CNC | NC | CN | CNC | CNC | CN |
| A0A3N6PYS5 | CNC | CNC | NC | CN | CNC | CNC | CN |
| A0A3P6GH01 | CNC | CNC | NC | CN | CNC | CNC | CN |
| A0A4D8YFY3 | CNC | CNC | NC | CN | CNC | CNC | CN |
| A0A565CCQ2 | CNC | CNC | NC | CN | CNC | CNC | CN |
| A0A5D2P151 | CNC | CNC | NC | CN | CNC | CNC | CN |
| A0A5D2XUL0 | CNC | CNC | NC | CN | CNC | CNC | CN |
| A0A5J5UEX2 | CNC | CNC | NC | CN | CNC | CNC | CN |

| | | | | | | | |
|---|---|---|---|---|---|---|---|
| A0A5N6RCL0 | CNC | CNC | NC | CN | CNC | CNC | CN |
| A0A5S9XWU8 | CNC | CNC | NC | CN | CNC | CNC | CN |
| A0A6C0QI02 | CNC | CNC | NC | CN | CNC | CNC | CN |
| A0A6D2IFA2 | CNC | CNC | NC | CN | CNC | CNC | CN |
| A0A6J0LWM4 | CNC | CNC | NC | CN | CNC | CNC | CN |
| A0A6J0MFM2 | CNC | CNC | NC | CN | CNC | CNC | CN |
| A0A7C8YCZ0 | CNC | CNC | NC | CN | CNC | CNC | CN |
| A0A816R937 | CNC | CNC | NC | CN | CNC | CNC | CN |
| A0A833QGX7 | CNC | CNC | NC | CN | CNC | CNC | CN |
| A0A835EWB9 | CNC | CNC | NC | CN | CNC | CNC | CN |
| A0A835TFK5 | CNC | CNC | NC | CN | CNC | CNC | CN |
| C3S7H4 | CNC | CNC | NC | CN | CNC | CNC | CN |
| C3S7H5 | CNC | CNC | NC | CN | CNC | CNC | CN |
| C3S7I2 | CNC | CNC | NC | CN | CNC | CNC | CN |
| D7MF58 | CNC | CNC | NC | CN | CNC | CNC | CN |
| M4EC98 | CNC | CNC | NC | CN | CNC | CNC | CN |
| Q0VH28 | CNC | CNC | NC | CN | CNC | CNC | CN |
| Q7XQ03 | CNC | CNC | NC | CN | CNC | CNC | CN |
| R0H2B9 | CNC | CNC | NC | CN | CNC | CNC | CN |
| V4MH41 | CNC | CNC | NC | CN | CNC | CNC | CN |
| A0A199UHE3 | CNC | CNC | NC | CN | CNC | CNC | CN |
| A0A3B6B301 | CNC | CNC | NC | CN | CNC | CNC | CN |
| A0A6P5ENU9 | CNC | CNC | NC | CN | CNC | CNC | CN |
| A0A8B7CKQ0 | CNC | CNC | NC | CN | CNC | CNC | CN |
| M8AVI7 | CNC | CNC | NC | CN | CNC | CNC | CN |
| A0A8J5YJS0 | CNC | CNC | NC | CN | CNC | CNC | CN |
| A0A1D6JTL2 | CNC | CNC | NC | CN | CNC | CNC | CN |
| A0A5P1E3V7 | CNC | CNC | NC | CN | CNC | CNC | CN |
| A0A0B0NEP1 | CNC | CNC | NC | CN | CNC | CNC | CN |
| A0A1U8HRH9 | CNC | CNC | NC | CN | CNC | CNC | CN |
| A0A5B6UCB8 | CNC | CNC | NC | CN | CNC | CNC | CN |
| A0A5D2F8A4 | CNC | CNC | NC | CN | CNC | CNC | CN |
| A0A5D2P2E3 | CNC | CNC | NC | CN | CNC | CNC | CN |
| A0A5D2XUU6 | CNC | CNC | NC | CN | CNC | CNC | CN |
| A0A5J5UAN9 | CNC | CNC | NC | CN | CNC | CNC | CN |
| A0A6A3ANX2 | CNC | CNC | NC | CN | CNC | CNC | CN |
| A0A835I2X3 | CNC | CNC | NC | CN | CNC | CNC | CN |
| A0A0D2STH6 | CNC | CNC | NC | CN | CNC | CNC | CN |
| A0A5D2B991 | CNC | CNC | NC | CN | CNC | CNC | CN |
| A0A5D2JFI3 | CNC | CNC | NC | CN | CNC | CNC | CN |
| A0A5D2TFS3 | CNC | CNC | NC | CN | CNC | CNC | CN |
| A0A5J5PZL4 | CNC | CNC | NC | CN | CNC | CNC | CN |
| A0A7J8RMG1 | CNC | CNC | NC | CN | CNC | CNC | CN |
| A0A7J9E2H7 | CNC | CNC | NC | CN | CNC | CNC | CN |
| C7J0T2 | CNC | CNC | NC | CN | CNC | CNC | CN |
| A0A8J5YQX7 | CNC | CNC | NC | CN | CNC | CNC | CN |
| R0GK29 | N | CNC | NC | CN | CNC | CNC | CN |

| | | | | | | | |
|---|---|---|---|---|---|---|---|
| A0A388KU01 | N | CNC | NC | CN | CNC | CNC | CN |
| A0A0B0PG83 | N | CNC | NC | CN | CNC | CNC | CN |
| A0A0D2Q514 | N | CNC | NC | CN | CNC | CNC | CN |
| A0A1D1YWY6 | N | CNC | NC | CN | CNC | CNC | CN |
| A0A1Q3CVR1 | N | CNC | NC | CN | CNC | CNC | CN |
| A0A1U8HZ48 | N | CNC | NC | CN | CNC | CNC | CN |
| A0A1U8I255 | N | CNC | NC | CN | CNC | CNC | CN |
| A0A251VQD4 | N | CNC | NC | CN | CNC | CNC | CN |
| A0A2P5YRJ0 | N | CNC | NC | CN | CNC | CNC | CN |
| A0A5B6UHK9 | N | CNC | NC | CN | CNC | CNC | CN |
| A0A5D2B6V9 | N | CNC | NC | CN | CNC | CNC | CN |
| A0A5D2F6L1 | N | CNC | NC | CN | CNC | CNC | CN |
| A0A5D2JE54 | N | CNC | NC | CN | CNC | CNC | CN |
| A0A5D2NZG1 | N | CNC | NC | CN | CNC | CNC | CN |
| A0A5D2TGD1 | N | CNC | NC | CN | CNC | CNC | CN |
| A0A5D2XV04 | N | CNC | NC | CN | CNC | CNC | CN |
| A0A5J5Q1V3 | N | CNC | NC | CN | CNC | CNC | CN |
| A0A5J9WGS9 | N | CNC | NC | CN | CNC | CNC | CN |
| A0A6J1ATY8 | N | CNC | NC | CN | CNC | CNC | CN |
| A0A6P5X2I3 | N | CNC | NC | CN | CNC | CNC | CN |
| A0A6P6A5V6 | N | CNC | NC | CN | CNC | CNC | CN |
| A0A7J8RNZ4 | N | CNC | NC | CN | CNC | CNC | CN |
| A0A7J8UJ26 | N | CNC | NC | CN | CNC | CNC | CN |
| A0A7J8ZLL1 | N | CNC | NC | CN | CNC | CNC | CN |
| A0A7J9BU30 | N | CNC | NC | CN | CNC | CNC | CN |
| A0A7J9E2E4 | N | CNC | NC | CN | CNC | CNC | CN |
| A0A7J9GQZ8 | N | CNC | NC | CN | CNC | CNC | CN |
| A0A7J9J722 | N | CNC | NC | CN | CNC | CNC | CN |
| A0A7J9LEI5 | N | CNC | NC | CN | CNC | CNC | CN |
| A0A067ERS3 | N | CNC | NC | CN | CNC | CNC | CN |
| A0A068TTC5 | N | CNC | NC | CN | CNC | CNC | CN |
| A0A0D2Q4Q5 | N | CNC | NC | CN | CNC | CNC | CN |
| A0A1R3JSA9 | N | CNC | NC | CN | CNC | CNC | CN |
| A0A200PVT7 | N | CNC | NC | CN | CNC | CNC | CN |
| A0A2K2CHF5 | N | CNC | NC | CN | CNC | CNC | CN |
| A0A2P5WJ73 | N | CNC | NC | CN | CNC | CNC | CN |
| A0A2P5X707 | N | CNC | NC | CN | CNC | CNC | CN |
| A0A3B6B331 | N | CNC | NC | CN | CNC | CNC | CN |
| A0A3L6PV57 | N | CNC | NC | CN | CNC | CNC | CN |
| A0A443Q0G6 | N | CNC | NC | CN | CNC | CNC | CN |
| A0A498I5B5 | N | CNC | NC | CN | CNC | CNC | CN |
| A0A4U5QPF5 | N | CNC | NC | CN | CNC | CNC | CN |
| A0A5D2B6R0 | N | CNC | NC | CN | CNC | CNC | CN |
| A0A5D2F8A1 | N | CNC | NC | CN | CNC | CNC | CN |
| A0A5D2JGD0 | N | CNC | NC | CN | CNC | CNC | CN |
| A0A5D2TIR9 | N | CNC | NC | CN | CNC | CNC | CN |
| A0A5J5PZZ1 | N | CNC | NC | CN | CNC | CNC | CN |

| | | | | | | | |
|---|---|---|---|---|---|---|---|
| A0A5J5UBE9 | N | CNC | NC | CN | CNC | CNC | CN |
| A0A5N5FAQ2 | N | CNC | NC | CN | CNC | CNC | CN |
| A0A6G1DIC8 | N | CNC | NC | CN | CNC | CNC | CN |
| A0A6J1AU56 | N | CNC | NC | CN | CNC | CNC | CN |
| A0A7I8IZQ3 | N | CNC | NC | CN | CNC | CNC | CN |
| A0A7I8KMB6 | N | CNC | NC | CN | CNC | CNC | CN |
| A0A7J7LPB0 | N | CNC | NC | CN | CNC | CNC | CN |
| A0A7N2L737 | N | CNC | NC | CN | CNC | CNC | CN |
| A0A811R1F3 | N | CNC | NC | CN | CNC | CNC | CN |
| A0A834H2X8 | N | CNC | NC | CN | CNC | CNC | CN |
| A0A835LRI6 | N | CNC | NC | CN | CNC | CNC | CN |
| A0A8J5VB55 | N | CNC | NC | CN | CNC | CNC | CN |
| A9NT86 | N | CNC | NC | CN | CNC | CNC | CN |
| A9NZJ2 | N | CNC | NC | CN | CNC | CNC | CN |
| B9HST3 | N | CNC | NC | CN | CNC | CNC | CN |
| C5YBZ6 | N | CNC | NC | CN | CNC | CNC | CN |
| D7SZN0 | N | CNC | NC | CN | CNC | CNC | CN |
| M5XS62 | N | CNC | NC | CN | CNC | CNC | CN |
| V4RIN0 | N | CNC | NC | CN | CNC | CNC | CN |
| W9QHH5 | N | CNC | NC | CN | CNC | CNC | CN |
| A0A078FXS4 | N | CNC | NC | CN | CNC | CNC | CN |
| A0A078G480 | N | CNC | NC | CN | CNC | CNC | CN |
| A0A2D1CIQ7 | N | CNC | NC | CN | CNC | CNC | CN |
| A0A2I0X983 | N | CNC | NC | CN | CNC | CNC | CN |
| A0A397ZCE5 | N | CNC | NC | CN | CNC | CNC | CN |
| A0A398A3A6 | N | CNC | NC | CN | CNC | CNC | CN |
| A0A3B6B2Z6 | N | CNC | NC | CN | CNC | CNC | CN |
| A0A3N6U5B1 | N | CNC | NC | CN | CNC | CNC | CN |
| A0A3P5Z444 | N | CNC | NC | CN | CNC | CNC | CN |
| A0A3P5ZII8 | N | CNC | NC | CN | CNC | CNC | CN |
| A0A3P6AAL2 | N | CNC | NC | CN | CNC | CNC | CN |
| A0A3P6BY39 | N | CNC | NC | CN | CNC | CNC | CN |
| A0A5B6UEG5 | N | CNC | NC | CN | CNC | CNC | CN |
| A0A5D2F857 | N | CNC | NC | CN | CNC | CNC | CN |
| A0A5D2NZC3 | N | CNC | NC | CN | CNC | CNC | CN |
| A0A5D2XXD2 | N | CNC | NC | CN | CNC | CNC | CN |
| A0A5J5UE99 | N | CNC | NC | CN | CNC | CNC | CN |
| A0A6D2IA45 | N | CNC | NC | CN | CNC | CNC | CN |
| A0A6J0JXU0 | N | CNC | NC | CN | CNC | CNC | CN |
| A0A6P6WXQ6 | N | CNC | NC | CN | CNC | CNC | CN |
| A0A816IAX4 | N | CNC | NC | CN | CNC | CNC | CN |
| A0A816V7Y2 | N | CNC | NC | CN | CNC | CNC | CN |
| D7LG96 | N | CNC | NC | CN | CNC | CNC | CN |
| M4CMR3 | N | CNC | NC | CN | CNC | CNC | CN |
| M4E2D6 | N | CNC | NC | CN | CNC | CNC | CN |
| A0A5J5C5H8 | N | CNC | NC | CN | CNC | CNC | CN |
| A0A5J6KLA9 | N | CNC | NC | CN | CNC | CNC | CN |

| | | | | | | | |
|---|---|---|---|---|---|---|---|
| A0A5P1EZB1 | N | CNC | NC | CN | CNC | CNC | CN |
| A0A6A3AJ13 | N | CNC | NC | CN | CNC | CNC | CN |
| A0A6P6A5U1 | N | CNC | NC | CN | CNC | CNC | CN |
| A0A6P6WZW0 | N | CNC | NC | CN | CNC | CNC | CN |
| A8I7R0 | N | CNC | NC | CN | CNC | CNC | CN |
| S8C3C9 | N | CNC | NC | CN | CNC | CNC | CN |
| A0A4S8K387 | N | CNC | NC | CN | CNC | CNC | CN |
| A0A0D9VRD3 | N | CNC | NC | CN | CNC | CNC | CN |
| A0A0U9HUL8 | N | CNC | NC | CN | CNC | CNC | CN |
| A0A1B5GEP3 | N | CNC | NC | CN | CNC | CNC | CN |
| A0A5P1EY07 | N | CNC | NC | CN | CNC | CNC | CN |
| A0A835LZ76 | N | CNC | NC | CN | CNC | CNC | CN |
| A0A843VQP8 | N | CNC | NC | CN | CNC | CNC | CN |
| A0A8I6XDR0 | N | CNC | NC | CN | CNC | CNC | CN |
| A0A8J5H632 | N | CNC | NC | CN | CNC | CNC | CN |
| A0A8J5HY48 | N | CNC | NC | CN | CNC | CNC | CN |
| C3VER2 | N | CNC | NC | CN | CNC | CNC | CN |
| F2EKR2 | N | CNC | NC | CN | CNC | CNC | CN |
| Q6UFY8 | N | CNC | NC | CN | CNC | CNC | CN |
| W5AQ78 | N | CNC | NC | CN | CNC | CNC | CN |
| B7FAF1 | N | CNC | NC | CN | CNC | CNC | CN |
| A0A067L8E5 | N | CNC | NC | CN | CNC | CNC | CN |
| A0A0D3FFL8 | N | CNC | NC | CN | CNC | CNC | CN |
| A0A0D9Z448 | N | CNC | NC | CN | CNC | CNC | CN |
| A0A0E0KAN7 | N | CNC | NC | CN | CNC | CNC | CN |
| A0A0E0NRS4 | N | CNC | NC | CN | CNC | CNC | CN |
| A0A150GA36 | N | CNC | NC | CN | CNC | CNC | CN |
| A0A1S4CDS3 | N | CNC | NC | CN | CNC | CNC | CN |
| A0A1U7WGZ2 | N | CNC | NC | CN | CNC | CNC | CN |
| A0A2G5CYB8 | N | CNC | NC | CN | CNC | CNC | CN |
| A0A2I0AHM6 | N | CNC | NC | CN | CNC | CNC | CN |
| A0A2I0XAW1 | N | CNC | NC | CN | CNC | CNC | CN |
| A0A2P5ANL5 | N | CNC | NC | CN | CNC | CNC | CN |
| A0A2P5FAL4 | N | CNC | NC | CN | CNC | CNC | CN |
| A0A2Z7BIX7 | N | CNC | NC | CN | CNC | CNC | CN |
| A0A2Z7BPH9 | N | CNC | NC | CN | CNC | CNC | CN |
| A0A314L536 | N | CNC | NC | CN | CNC | CNC | CN |
| A0A498HPC0 | N | CNC | NC | CN | CNC | CNC | CN |
| A0A5N5HHW6 | N | CNC | NC | CN | CNC | CNC | CN |
| A0A6A3D4V4 | N | CNC | NC | CN | CNC | CNC | CN |
| A0A6A6K9Q6 | N | CNC | NC | CN | CNC | CNC | CN |
| A0A6G1EDZ7 | N | CNC | NC | CN | CNC | CNC | CN |
| A0A804K5T6 | N | CNC | NC | CN | CNC | CNC | CN |
| A0A834HEJ1 | N | CNC | NC | CN | CNC | CNC | CN |
| A0A8B8N2Q8 | N | CNC | NC | CN | CNC | CNC | CN |
| I1H7Y6 | N | CNC | NC | CN | CNC | CNC | CN |
| A0A2C9W9G2 | N | CNC | NC | CN | CNC | CNC | CN |

| | | | | | | | |
|---|---|---|---|---|---|---|---|
| A0A453CF70 | N | CNC | NC | CN | CNC | CNC | CN |
| A0A4Y1QLB4 | N | CNC | NC | CN | CNC | CNC | CN |
| A0A453CFB7 | N | CNC | NC | CN | CNC | CNC | CN |
| A0A0A9VG64 | N | CNC | NC | CN | CNC | CNC | CN |
| A0A0D9VRD2 | N | CNC | NC | CN | CNC | CNC | CN |
| A0A368STT0 | N | CNC | NC | CN | CNC | CNC | CN |
| A0A4U6T993 | N | CNC | NC | CN | CNC | CNC | CN |
| A0A1U8HRI4 | N | CNC | NC | CN | CNC | CNC | CN |
| A0A6P4LQL4 | N | CNC | NC | CN | CNC | CNC | CN |
| A0A5B6UCE2 | N | CNC | NC | CN | CNC | CNC | CN |
| A0A5D2NZG9 | N | CNC | NC | CN | CNC | CNC | CN |
| A0A5D2XU78 | N | CNC | NC | CN | CNC | CNC | CN |
| A0A6A2Z6K5 | N | CNC | NC | CN | CNC | CNC | CN |
| A0A0B0NDJ6 | N | CNC | NC | CN | CNC | CNC | CN |
| A0A1U8I7W9 | N | CNC | NC | CN | CNC | CNC | CN |
| A0A2P5X709 | N | CNC | NC | CN | CNC | CNC | CN |
| A0A7J9IEV2 | N | CNC | NC | CN | CNC | CNC | CN |
| A0A8J5YMW0 | N | CNC | NC | CN | CNC | CNC | CN |
| A0A1Z5SAN6 | N | CNC | NC | CN | CNC | CNC | CN |
| A0A2S3I1C8 | N | CNC | NC | CN | CNC | CNC | CN |
| A0A2T7D5B0 | N | CNC | NC | CN | CNC | CNC | CN |
| A0A5J9WIK7 | N | CNC | NC | CN | CNC | CNC | CN |
| A0A6A2Z2D2 | N | CNC | NC | CN | CNC | CNC | CN |
| A0A6A4LJP3 | N | CNC | NC | CN | CNC | CNC | CN |
| A0A6P5GBE9 | NC | CNC | NC | CN | CNC | CNC | CN |
| A0A0A0LLG2 | NC | CNC | NC | CN | CNC | CNC | CN |
| A0A0D3B4P8 | NC | CNC | NC | CN | CNC | CNC | CN |
| A0A251U3E7 | NC | CNC | NC | CN | CNC | CNC | CN |
| A0A2G9I125 | NC | CNC | NC | CN | CNC | CNC | CN |
| A0A2J6K6G2 | NC | CNC | NC | CN | CNC | CNC | CN |
| A0A2K3DNH3 | NC | CNC | NC | CN | CNC | CNC | CN |
| A0A4D9CUG0 | NC | CNC | NC | CN | CNC | CNC | CN |
| A0A5D3B9W9 | NC | CNC | NC | CN | CNC | CNC | CN |
| A0A5N6QS25 | NC | CNC | NC | CN | CNC | CNC | CN |
| A0A835RN30 | NC | CNC | NC | CN | CNC | CNC | CN |
| K7TUS9 | NC | CNC | NC | CN | CNC | CNC | CN |
| A0A1U9ZCC4 | NC | CNC | NC | CN | CNC | CNC | CN |
| A0A2P5FYN8 | NC | CNC | NC | CN | CNC | CNC | CN |
| A0A2Z6LW86 | NC | CNC | NC | CN | CNC | CNC | CN |
| A0A444XQT1 | NC | CNC | NC | CN | CNC | CNC | CN |
| A0A445BHD3 | NC | CNC | NC | CN | CNC | CNC | CN |
| A0A5N5L4L9 | NC | CNC | NC | CN | CNC | CNC | CN |
| A0A699L3M0 | NC | CNC | NC | CN | CNC | CNC | CN |
| A0A6M2EY82 | NC | CNC | NC | CN | CNC | CNC | CN |
| A0A6P4BJA6 | NC | CNC | NC | CN | CNC | CNC | CN |
| A0A6S7M4D2 | NC | CNC | NC | CN | CNC | CNC | CN |
| A0A7J6F280 | NC | CNC | NC | CN | CNC | CNC | CN |

| | | | | | | | |
|---|---|---|---|---|---|---|---|
| A0A836Y2K8 | NC | CNC | NC | CN | CNC | CNC | CN |
| A0A0D9ZN25 | NC | CNC | NC | CN | CNC | CNC | CN |
| A0A0E0KSQ4 | NC | CNC | NC | CN | CNC | CNC | CN |
| A0A0K9PK88 | NC | CNC | NC | CN | CNC | CNC | CN |
| A0A1R3JSQ3 | NC | CNC | NC | CN | CNC | CNC | CN |
| A0A2P5FAM1 | NC | CNC | NC | CN | CNC | CNC | CN |
| A0A5A7U8N2 | NC | CNC | NC | CN | CNC | CNC | CN |
| A0A6G1EE11 | NC | CNC | NC | CN | CNC | CNC | CN |
| A0A6J1C9H8 | NC | CNC | NC | CN | CNC | CNC | CN |
| A0A6J1H408 | NC | CNC | NC | CN | CNC | CNC | CN |
| A0A6J1L161 | NC | CNC | NC | CN | CNC | CNC | CN |
| A0A7N0V868 | NC | CNC | NC | CN | CNC | CNC | CN |
| B0F824 | NC | CNC | NC | CN | CNC | CNC | CN |
| J3LZI1 | NC | CNC | NC | CN | CNC | CNC | CN |
| A0A6G1DIY3 | NC | CNC | NC | CN | CNC | CNC | CN |
| A0A2I4DSD4 | NC | CNC | NC | CN | CNC | CNC | CN |
| A0A059B288 | NC | CNC | NC | CN | CNC | CNC | CN |
| A0A068TSS8 | NC | CNC | NC | CN | CNC | CNC | CN |
| A0A0D9W726 | NC | CNC | NC | CN | CNC | CNC | CN |
| A0A0E0DG21 | NC | CNC | NC | CN | CNC | CNC | CN |
| A0A1U8APS2 | NC | CNC | NC | CN | CNC | CNC | CN |
| A0A1Y1HLG0 | NC | CNC | NC | CN | CNC | CNC | CN |
| A0A218WEP3 | NC | CNC | NC | CN | CNC | CNC | CN |
| A0A5J5AC15 | NC | CNC | NC | CN | CNC | CNC | CN |
| A0A6N2N1M0 | NC | CNC | NC | CN | CNC | CNC | CN |
| A0A6P6WTQ2 | NC | CNC | NC | CN | CNC | CNC | CN |
| A0A6P6X235 | NC | CNC | NC | CN | CNC | CNC | CN |
| A0A7J7E1D1 | NC | CNC | NC | CN | CNC | CNC | CN |
| A0A7N0V563 | NC | CNC | NC | CN | CNC | CNC | CN |
| A0A830BHP1 | NC | CNC | NC | CN | CNC | CNC | CN |
| A0A834HF62 | NC | CNC | NC | CN | CNC | CNC | CN |
| A0A6P5G915 | NC | CNC | NC | CN | CNC | CNC | CN |
| A0A7J0GIR6 | NC | CNC | NC | CN | CNC | CNC | CN |
| A0A7J7LPK4 | CNC | CN | CN | CNC | CNC | CNC | CN |
| A0A1E5VQ43 | CNC | CNC | CN | CNC | CNC | CNC | CN |
| A0A7H4LKC5 | CN | CNC | NC | CNC | CNC | CNC | CN |
| A0A1D6JTL4 | CNC | CNC | NC | CNC | CNC | CNC | CN |
| A0A317YHZ9 | CNC | CNC | NC | CNC | CNC | CNC | CN |
| B6U6A9 | CNC | CNC | NC | CNC | CNC | CNC | CN |
| A0A5J9WIV0 | CNC | CNC | NC | CNC | CNC | CNC | CN |
| D8TIC1 | CNC | CNC | NC | CNC | CNC | CNC | CN |
| A0A150GQQ2 | CNC | CNC | NC | CNC | CNC | CNC | CN |
| A0A5N6P4A4 | CNC | CNC | NC | CNC | CNC | CNC | CN |
| A0A835YFW8 | CNC | CNC | NC | CNC | CNC | CNC | CN |
| A0A836B1Z6 | CNC | CNC | NC | CNC | CNC | CNC | CN |
| A0A843XT84 | CNC | CNC | NC | CNC | CNC | CNC | CN |
| A0A8J4BEF7 | CNC | CNC | NC | CNC | CNC | CNC | CN |

| | | | | | | | |
|---|---|---|---|---|---|---|---|
| A0A5N6M8N9 | CN | CNC | NC | N | CNC | CNC | CN |
| A0A0D2UUL3 | N | CNC | NC | N | CNC | CNC | CN |
| A0A1U8IS89 | N | CNC | NC | N | CNC | CNC | CN |
| A0A2J6K682 | N | CNC | NC | N | CNC | CNC | CN |
| A0A5B6WGA0 | N | CNC | NC | N | CNC | CNC | CN |
| A0A5D2B035 | N | CNC | NC | N | CNC | CNC | CN |
| A0A5D2EY41 | N | CNC | NC | N | CNC | CNC | CN |
| A0A5D2NPB4 | N | CNC | NC | N | CNC | CNC | CN |
| A0A5D2TBU6 | N | CNC | NC | N | CNC | CNC | CN |
| A0A5D2XKM1 | N | CNC | NC | N | CNC | CNC | CN |
| A0A5J5PT10 | N | CNC | NC | N | CNC | CNC | CN |
| A0A5J5U2E2 | N | CNC | NC | N | CNC | CNC | CN |
| A0A6P4MFJ7 | N | CNC | NC | N | CNC | CNC | CN |
| A0A7J8N1E7 | N | CNC | NC | N | CNC | CNC | CN |
| A0A7J8SSI4 | N | CNC | NC | N | CNC | CNC | CN |
| A0A7J8YBX6 | N | CNC | NC | N | CNC | CNC | CN |
| A0A7J9HTK2 | N | CNC | NC | N | CNC | CNC | CN |
| A0A7J9K455 | N | CNC | NC | N | CNC | CNC | CN |
| A0A7J9MIZ7 | N | CNC | NC | N | CNC | CNC | CN |
| A0A0D2VNL5 | N | CNC | NC | N | CNC | CNC | CN |
| A0A1U8IK60 | N | CNC | NC | N | CNC | CNC | CN |
| A0A1U8KDP9 | N | CNC | NC | N | CNC | CNC | CN |
| A0A5D2B233 | N | CNC | NC | N | CNC | CNC | CN |
| A0A5D2EXT0 | N | CNC | NC | N | CNC | CNC | CN |
| A0A5D2NPE0 | N | CNC | NC | N | CNC | CNC | CN |
| A0A5D2T8C2 | N | CNC | NC | N | CNC | CNC | CN |
| A0A5D2XKN8 | N | CNC | NC | N | CNC | CNC | CN |
| A0A5J5PU59 | N | CNC | NC | N | CNC | CNC | CN |
| A0A5J5U2J9 | N | CNC | NC | N | CNC | CNC | CN |
| A0A6P4MSZ9 | N | CNC | NC | N | CNC | CNC | CN |
| A0A7J8SSE4 | N | CNC | NC | N | CNC | CNC | CN |
| A0A7J8YAC2 | N | CNC | NC | N | CNC | CNC | CN |
| A0A7J9HTJ4 | N | CNC | NC | N | CNC | CNC | CN |
| W9QSJ4 | N | CNC | NC | N | CNC | CNC | CN |
| A0A3N6SVI9 | N | CNC | NC | N | CNC | CNC | CN |
| A0A1B5GEW3 | N | CNC | NC | N | CNC | CNC | CN |
| A0A3B6JKY4 | N | CNC | NC | N | CNC | CNC | CN |
| A0A453IL99 | N | CNC | NC | N | CNC | CNC | CN |
| A0A453ILR7 | N | CNC | NC | N | CNC | CNC | CN |
| M8ATL3 | N | CNC | NC | N | CNC | CNC | CN |
| X4YJY3 | N | CNC | NC | N | CNC | CNC | CN |
| A0A2Z7CSS8 | N | CNC | NC | N | CNC | CNC | CN |
| A0A6A2Z101 | N | CNC | NC | N | CNC | CNC | CN |
| A0A6A2Z6K6 | N | CNC | NC | N | CNC | CNC | CN |
| A0A6J0LAF8 | N | CNC | NC | N | CNC | CNC | CN |
| A0A835Y138 | N | CNC | NC | N | CNC | CNC | CN |
| A0A0E0DG19 | N | CNC | NC | N | CNC | CNC | CN |

| | | | | | | | |
|---|---|---|---|---|---|---|---|
| A0A453IM18 | N | CNC | NC | N | CNC | CNC | CN |
| A0A0E0NRS3 | N | CNC | NC | N | CNC | CNC | CN |
| Q10PT1 | N | CNC | NC | N | CNC | CNC | CN |
| A0A3B6HS90 | N | CNC | NC | N | CNC | CNC | CN |
| A0A3B6HT46 | N | CNC | NC | N | CNC | CNC | CN |
| A0A3B6IVB0 | N | CNC | NC | N | CNC | CNC | CN |
| A0A3B6IW03 | N | CNC | NC | N | CNC | CNC | CN |
| A0A8I6XMJ1 | N | CNC | NC | N | CNC | CNC | CN |
| A0A0D9Z447 | N | CNC | NC | N | CNC | CNC | CN |
| A0A0E0CL18 | N | CNC | NC | N | CNC | CNC | CN |
| A0A0E0HJX6 | N | CNC | NC | N | CNC | CNC | CN |
| A0A811MA55 | N | CNC | NC | N | CNC | CNC | CN |
| A0A835AVD9 | N | CNC | NC | N | CNC | CNC | CN |
| I1P911 | N | CNC | NC | N | CNC | CNC | CN |
| A0A6A2Z1B5 | N | CNC | NC | N | CNC | CNC | CN |
| A0A6A2Z2A1 | N | CNC | NC | N | CNC | CNC | CN |
| A0A453ILQ5 | N | CNC | NC | N | CNC | CNC | CN |
| A0A6A3D0Q0 | N | CNC | NC | N | CNC | CNC | CN |
| A0A7J7Q6V3 | N | CNC | NC | N | CNC | CNC | CN |
| A0A0D6RA28 | NC | CNC | NC | N | CNC | CNC | CN |
| A0A6G1DJC3 | NC | CNC | NC | N | CNC | CNC | CN |
| A0A835WI91 | N | CNC | NC | NCN | CNC | CNC | CN |
| A0A7J7PIC5 | N | N | NC | N | N | CNC | CN |
| E1ZMG0 | CN | CN | CN | CN | NC | CNC | CN |
| A0A1J3DE81 | CN | CNC | NC | CN | NC | CNC | CN |
| A0A2J8A1U4 | N | CNC | NC | CN | NC | CNC | CN |
| K4AE37 | N | CNC | NC | CN | NC | CNC | CN |
| A0A2R6PVY3 | N | CNC | NC | CN | NC | CNC | CN |
| A0A1D1ZU75 | N | CNC | NC | N | NC | CNC | CN |
| A0A0D9VRD1 | N | NCN | NC | N | NCN | CNC | CN |
| A0A0E0KAN8 | CN | CNC | NC | CN | CNC | NC | CN |
| A0A0Q3SCX9 | N | CNC | NC | N | CNC | NC | CN |
| A0A0E0E020 | N | CN | CN | CN | NC | NC | CN |
| A0A3N6R4W9 | NC | CN | CN | CN | NC | NC | CN |
| A0A565AZP9 | NC | CN | CN | CN | NC | NC | CN |
| A0A835NAP7 | NC | NC | NC | N | NC | NC | CN |
| A0A833R129 | CNC | CN | NCN | CNC | CN | CN | CNC |
| A0A060L102 | NCN | CNC | CNC | CNC | CNC | CN | CNC |
| A0A445LGT1 | CNC | CNC | NCN | CNC | CNC | CN | CNC |
| A0A1I9R3Y6 | NCN | CNC | CNC | NC | CNC | CN | CNC |
| A0A060L4I9 | NCN | CNC | CNC | CNC | NC | CN | CNC |
| A0A453FGB9 | N | CNC | CNC | CN | CN | CNC | CNC |
| A0A835EZD4 | CNC | CNC | CNC | CNC | CN | CNC | CNC |
| A0A804LQL5 | CNC | CNC | CNC | CNC | CN | CNC | CNC |
| A0A804LQL6 | CNC | CNC | CNC | CNC | CN | CNC | CNC |
| A0A453RA17 | CN | CNC | CNC | N | CN | CNC | CNC |
| A0A0D3GEY9 | N | CNC | CNC | N | CN | CNC | CNC |

| | | | | | | | |
|---|---|---|---|---|---|---|---|
| A0A4U6UZE3 | N | CNC | CNC | N | CN | CNC | CNC |
| K3XYS1 | N | CNC | CNC | N | CN | CNC | CNC |
| A0A2U1PSY4 | CNC | CNC | NCN | CN | CNC | CNC | CNC |
| A0A388KU64 | N | CNC | NCN | CN | CNC | CNC | CNC |
| A0A0D3FA60 | CN | CNC | CNC | CNC | CNC | CNC | CNC |
| A0A4U6V230 | CNC | CNC | CNC | CNC | CNC | CNC | CNC |
| K3YU83 | CNC | CNC | CNC | CNC | CNC | CNC | CNC |
| A0A453PI55 | N | CNC | CNC | CNC | CNC | CNC | CNC |
| A0A6A6UK47 | CNC | CNC | NCN | CNC | CNC | CNC | CNC |
| A0A6A4PUY9 | CNC | CNC | NCN | CNC | CNC | CNC | CNC |
| A0A6A4PV32 | N | CNC | CNC | CNC | NC | CNC | CNC |
| A0A811SG28 | N | NC | CNC | N | NC | CNC | CNC |
| N1QYT1 | N | CNC | CNC | CN | CN | NC | CNC |
| A0A5P1FAC5 | NC | CNC | NCN | CNC | CNC | NC | CNC |
| A0A061EME7 | CNC | CNC | CNC | CN | NC | NC | CNC |
| A0A443N967 | NC | NC | CNC | NC | NC | NC | CNC |
| A0A1D6Q9V1 | N | N | CN | N | N | CN | N |
| A0A0E0LA25 | N | N | CN | N | N | CN | N |
| A0A3L6DAW3 | N | N | N | N | N | CN | N |
| A0A1D6IYI8 | N | N | NC | N | N | CN | N |
| A2X9B9 | N | N | CN | N | N | CNC | N |
| A0A835EZH0 | CN | CN | N | CN | CN | N | N |
| A0A0E0PVY0 | N | CN | N | CN | CN | N | N |
| A0A453RA96 | NC | CN | N | CN | CN | N | N |
| A0A0E0PVX6 | CN | N | N | CN | CN | N | N |
| A0A7J0G5S3 | CN | N | N | CN | CN | N | N |
| A0A2Z7C029 | CN | N | N | CN | CN | N | N |
| A0A453R9H9 | CN | N | N | CN | CN | N | N |
| A0A453RA56 | CN | N | N | CN | CN | N | N |
| A0A1U8B5C6 | CNC | N | N | CN | CN | N | N |
| A0A453RA58 | N | N | N | CN | CN | N | N |
| A0A368PR35 | N | CN | N | N | CN | N | N |
| A0A199VNQ9 | N | CN | N | N | CN | N | N |
| A0A5C7HLG7 | CN | N | N | N | CN | N | N |
| A0A835LX71 | N | N | N | N | CN | N | N |
| A0A5D2J6W3 | N | N | N | N | CN | N | N |
| A0A453PIB4 | N | N | N | N | CN | N | N |
| A0A2U1NM85 | N | N | N | N | CN | N | N |
| A0A0A9BVF3 | N | N | N | N | CN | N | N |
| A0A0E0ISE2 | N | N | N | N | CN | N | N |
| A0A2J6LWZ1 | N | N | N | N | CN | N | N |
| A0A453CF98 | N | N | N | N | CN | N | N |
| A0A5N6QT25 | N | N | N | N | CN | N | N |
| A0A4S8LF28 | N | N | N | N | CN | N | N |
| A0A484NMG8 | N | N | N | N | CN | N | N |
| A0A1U8I244 | CN | N | N | CN | CNC | N | N |
| A0A5D2TIV9 | CN | N | N | CN | CNC | N | N |

| | | | | | | | |
|---|---|---|---|---|---|---|---|
| A0A2G2W1M8 | CNC | N | N | CN | CNC | N | N |
| A0A2G2ZU95 | CNC | N | N | CN | CNC | N | N |
| A0A2G3CR31 | CNC | N | N | CN | CNC | N | N |
| A0A3B6CC07 | CNC | N | N | CN | CNC | N | N |
| A0A7J0F3C3 | NC | N | N | CN | CNC | N | N |
| M1DY77 | NC | N | N | CN | CNC | N | N |
| A0A8B7BGI3 | N | CN | N | CNC | CNC | N | N |
| A0A833VLL3 | CN | N | N | CNC | CNC | N | N |
| A0A453RA65 | CN | N | N | CNC | CNC | N | N |
| A0A8J4DAW7 | CNC | CN | N | N | CNC | N | N |
| A0A2J8AH52 | N | CN | N | N | CNC | N | N |
| A0A371FSK0 | N | N | N | N | CNC | N | N |
| A0A6A3D5Q1 | N | N | N | N | CNC | N | N |
| A0A811MLI9 | N | N | N | N | CNC | N | N |
| A0A8J5YDZ8 | N | N | N | N | CNC | N | N |
| R0ET62 | N | N | N | N | CNC | N | N |
| A0A118K4D0 | N | N | N | N | CNC | N | N |
| A0A199W1G2 | N | N | N | N | CNC | N | N |
| A0A445F7S1 | N | N | N | N | CNC | N | N |
| A0A4D8ZW70 | N | N | N | N | CNC | N | N |
| A0A7J6F281 | N | N | N | N | CNC | N | N |
| A0A8B9APT3 | N | N | N | N | CNC | N | N |
| A3AFL8 | N | N | N | N | CNC | N | N |
| A0A0E0FU55 | N | N | N | N | CNC | N | N |
| A0A0D9VKP6 | N | N | N | N | CNC | N | N |
| A0A0E0KSQ3 | N | N | N | N | CNC | N | N |
| A0A7J8N1D3 | N | N | N | N | CNC | N | N |
| A0A1U8HVH1 | N | N | N | N | CNC | N | N |
| A0A0D3FXV0 | N | N | N | N | CNC | N | N |
| A0A1Y1INH0 | N | N | N | N | CNC | N | N |
| A0A3B6TGP6 | NC | N | N | N | CNC | N | N |
| M8BWM1 | NC | N | N | N | CNC | N | N |
| A0A5C7IA63 | NCN | N | N | N | CNC | N | N |
| A0A8J4CHT0 | N | N | N | NC | CNC | N | N |
| A0A1D1ZET5 | N | N | N | CN | N | N | N |
| A0A835HUY5 | N | N | N | N | N | N | N |
| A0A0E0NKM2 | N | N | N | N | N | N | N |
| A0A3N7FQR1 | N | N | N | N | N | N | N |
| A0A803P498 | N | N | N | N | N | N | N |
| A0A2I0ARY8 | N | N | N | N | N | N | N |
| A0A453RA51 | N | N | N | N | N | N | N |
| A0A453RA82 | N | N | N | N | N | N | N |
| A0A453RA87 | N | N | N | N | N | N | N |
| A0A5D2GMR8 | N | N | N | N | N | N | N |
| A0A7J7GMB0 | N | N | N | N | N | N | N |
| A0A2U1Q6Y0 | N | N | N | N | N | N | N |
| A0A5B7CB08 | N | N | N | N | N | N | N |

| | | | | | | | |
|---|---|---|---|---|---|---|---|
| A0A0R0EDQ8 | N | N | N | N | N | N | N |
| A0A1R3JGT3 | N | N | N | N | N | N | N |
| A0A1R3JSK6 | N | N | N | N | N | N | N |
| A0A453CEU9 | N | N | N | N | N | N | N |
| A0A453R9L8 | N | N | N | N | N | N | N |
| A0A5J5B8N1 | N | N | N | N | N | N | N |
| A0A7J8ST91 | N | N | N | N | N | N | N |
| A0A7J9AMF2 | N | N | N | N | N | N | N |
| A0A7J9CM91 | N | N | N | N | N | N | N |
| A0A7J9MJE1 | N | N | N | N | N | N | N |
| A0A7J9MLG3 | N | N | N | N | N | N | N |
| A0A804PRC7 | N | N | N | N | N | N | N |
| A0A5D2L2I5 | N | N | N | N | N | N | N |
| A0A6A2YDU0 | N | N | N | N | N | N | N |
| A0A1J6L6C3 | N | N | N | N | N | N | N |
| A0A251VQJ3 | N | N | N | N | N | N | N |
| A0A445C7P5 | N | N | N | N | N | N | N |
| A0A699KJB3 | N | N | N | N | N | N | N |
| A0A7J6DZT9 | N | N | N | N | N | N | N |
| A0A834TVT6 | N | N | N | N | N | N | N |
| A0A022RXJ6 | N | N | N | N | N | N | N |
| A0A2U1Q6M8 | N | N | N | N | N | N | N |
| A0A453CIM5 | N | N | N | N | N | N | N |
| A0A5D2QQK6 | N | N | N | N | N | N | N |
| A0A7J9E3M2 | N | N | N | N | N | N | N |
| A0A8J5GUA7 | N | N | N | N | N | N | N |
| A0A5K0YQH6 | N | N | N | N | N | N | N |
| A0A5K1FVI0 | N | N | N | N | N | N | N |
| A0A5K1FZK7 | N | N | N | N | N | N | N |
| A0A835KF13 | N | N | N | N | N | N | N |
| M8CN42 | N | N | N | N | N | N | N |
| A0A453RA48 | N | N | N | N | N | N | N |
| A0A4D6LMA5 | N | N | N | N | N | N | N |
| A0A811RB71 | N | N | N | N | N | N | N |
| A0A0E0A7R6 | N | N | N | N | N | N | N |
| A0A7J8QEN2 | N | N | N | N | N | N | N |
| A0A7J8UIP7 | N | N | N | N | N | N | N |
| A0A7J8VQI2 | N | N | N | N | N | N | N |
| A0A7J9CMJ5 | N | N | N | N | N | N | N |
| A3BAC3 | N | N | N | N | N | N | N |
| B8B0A4 | N | N | N | N | N | N | N |
| M7YAJ2 | N | N | N | N | N | N | N |
| A0A0E0HNK9 | N | N | N | N | N | N | N |
| A0A1S3DYV3 | N | N | N | N | N | N | N |
| A0A2P5ABD4 | N | N | N | N | N | N | N |
| A0A2P5BG68 | N | N | N | N | N | N | N |
| A0A368QT22 | N | N | N | N | N | N | N |

| | | | | | | | |
|---|---|---|---|---|---|---|---|
| A0A3L6EKF7 | N | N | N | N | N | N | N |
| A0A3N6RHZ2 | N | N | N | N | N | N | N |
| A0A453NLW0 | N | N | N | N | N | N | N |
| A0A453RA91 | N | N | N | N | N | N | N |
| A0A5J9UYN5 | N | N | N | N | N | N | N |
| A0A7J8T8G4 | N | N | N | N | N | N | N |
| A0A7J9FH67 | N | N | N | N | N | N | N |
| A0A811S7F6 | N | N | N | N | N | N | N |
| A0A811SGE0 | N | N | N | N | N | N | N |
| A0A835D454 | N | N | N | N | N | N | N |
| A0A835KC15 | N | N | N | N | N | N | N |
| A0A835MQH6 | N | N | N | N | N | N | N |
| A2X9B7 | N | N | N | N | N | N | N |
| M8A7D6 | N | N | N | N | N | N | N |
| V7C7L9 | N | N | N | N | N | N | N |
| A0A444FSG4 | N | N | N | N | N | N | N |
| A0A7J8X960 | N | N | N | N | N | N | N |
| A0A7J9BU01 | N | N | N | N | N | N | N |
| A0A7J9J897 | N | N | N | N | N | N | N |
| A0A7J9LFZ8 | N | N | N | N | N | N | N |
| A0A803PCS3 | N | N | N | N | N | N | N |
| A0A0D3FA58 | N | N | N | N | N | N | N |
| A0A0D3FA59 | N | N | N | N | N | N | N |
| A0A1D6JTK6 | N | N | N | N | N | N | N |
| A0A1E5VQ98 | N | N | N | N | N | N | N |
| A0A2Z6NFB9 | N | N | N | N | N | N | N |
| A0A453ILS2 | N | N | N | N | N | N | N |
| A0A835I1H8 | N | N | N | N | N | N | N |
| Q45RI1 | N | N | N | N | N | N | N |
| A0A0D3GEZ1 | N | N | N | N | N | N | N |
| A0A1D6JTK9 | N | N | N | N | N | N | N |
| A0A1U7VG78 | N | N | N | N | N | N | N |
| A0A3L6EK75 | N | N | N | N | N | N | N |
| A0A3L6RWP7 | N | N | N | N | N | N | N |
| A0A445BZG1 | N | N | N | N | N | N | N |
| A0A453CEW9 | N | N | N | N | N | N | N |
| A0A453CF72 | N | N | N | N | N | N | N |
| A0A5D2CRK8 | N | N | N | N | N | N | N |
| A0A5K1FSW7 | N | N | N | N | N | N | N |
| A0A7J7GX31 | N | N | N | N | N | N | N |
| A0A7J8UIL8 | N | N | N | N | N | N | N |
| A0A7J8W3E3 | N | N | N | N | N | N | N |
| A0A7J9BTZ7 | N | N | N | N | N | N | N |
| A0A7J9BU87 | N | N | N | N | N | N | N |
| A0A7J9J5T7 | N | N | N | N | N | N | N |
| A0A7J9J6B5 | N | N | N | N | N | N | N |
| A0A7J9LHH6 | N | N | N | N | N | N | N |

| | | | | | | | |
|---|---|---|---|---|---|---|---|
| A0A811S6L7 | N | N | N | N | N | N | N |
| A0A835KDF1 | N | N | N | N | N | N | N |
| A0A5K0YS91 | N | N | N | N | N | N | N |
| A0A5K1FWA3 | N | N | N | N | N | N | N |
| D6PW68 | N | N | N | N | N | N | N |
| A0A5J9TKI2 | N | N | N | N | N | N | N |
| A0A2S3HKM6 | N | N | N | N | N | N | N |
| A0A2T7E1A8 | N | N | N | N | N | N | N |
| A0A3B6NUJ7 | N | N | N | N | N | N | N |
| A0A834ZUI4 | N | N | N | N | N | N | N |
| A0A8I7BHE5 | N | N | N | N | N | N | N |
| M8AN17 | N | N | N | N | N | N | N |
| A0A7J9J5P2 | N | N | N | N | N | N | N |
| A0A7J9KEQ9 | N | N | N | N | N | N | N |
| A0A3L6F198 | N | N | N | N | N | N | N |
| A0A453RA38 | N | N | N | N | N | N | N |
| A0A1D6JTL5 | N | N | N | N | N | N | N |
| A0A5C7HJH2 | N | N | N | N | N | N | N |
| A0A7J8RMD3 | N | N | N | N | N | N | N |
| A0A0E0E018 | N | N | N | N | N | N | N |
| A0A3N6QPJ1 | N | N | N | N | N | N | N |
| A0A453CEU6 | N | N | N | N | N | N | N |
| A0A453FG73 | N | N | N | N | N | N | N |
| A0A1R3G636 | N | N | N | N | N | N | N |
| A0A1R3JN15 | N | N | N | N | N | N | N |
| A0A5K0YSN0 | N | N | N | N | N | N | N |
| A0A5K0YSV5 | N | N | N | N | N | N | N |
| A0A5K1FT15 | N | N | N | N | N | N | N |
| A0A5K1FW66 | N | N | N | N | N | N | N |
| A0A6A5NY73 | N | N | N | N | N | N | N |
| A0A0E0HNK6 | N | N | N | N | N | N | N |
| A3BAC4 | N | N | N | N | N | N | N |
| B3SL20 | N | N | N | N | N | N | N |
| A0A5K1FT94 | N | N | N | N | N | N | N |
| A0A5K1FXW4 | N | N | N | N | N | N | N |
| A0A453CF91 | N | N | N | N | N | N | N |
| A0A2K3LUN0 | N | N | N | N | N | N | N |
| G7KML4 | N | N | N | N | N | N | N |
| A0A443N960 | N | N | N | N | N | N | N |
| A0A453NKH5 | N | N | N | N | N | N | N |
| A0A453NKL7 | N | N | N | N | N | N | N |
| A0A453RA45 | N | N | N | N | N | N | N |
| A0A453RA80 | N | N | N | N | N | N | N |
| A0A4D9BQS1 | N | N | N | N | N | N | N |
| A0A4S4DA53 | N | N | N | N | N | N | N |
| A0A5J5B6X1 | N | N | N | N | N | N | N |
| A0A6J0LLB2 | N | N | N | N | N | N | N |

| | | | | | | | |
|---|---|---|---|---|---|---|---|
| A0A7J9H765 | N | N | N | N | N | N | N |
| M7YIR8 | N | N | N | N | N | N | N |
| A0A811SAI2 | N | N | N | N | N | N | N |
| A0A427AQA4 | N | N | N | N | N | N | N |
| A0A2U1NM68 | N | N | N | N | N | N | N |
| A0A7J6WA85 | N | N | N | N | N | N | N |
| A0A843WWQ7 | N | N | N | N | N | N | N |
| A0A067FAX3 | N | N | N | N | N | N | N |
| A0A6A4R0R4 | N | N | N | N | N | N | N |
| A0A453PI45 | N | N | N | N | N | N | N |
| A0A6A3CBP4 | N | N | N | N | N | N | N |
| A0A6A6MWN1 | N | N | N | N | N | N | N |
| M1AL40 | N | N | N | N | N | N | N |
| A0A1R3G6B0 | N | N | N | N | N | N | N |
| A0A1U8I1D1 | N | N | N | N | N | N | N |
| A0A2P5ANM8 | N | N | N | N | N | N | N |
| A0A2P5DAK9 | N | N | N | N | N | N | N |
| A0A2P5FAL8 | N | N | N | N | N | N | N |
| A0A453CF78 | N | N | N | N | N | N | N |
| A0A453CIN2 | N | N | N | N | N | N | N |
| A0A453CJ11 | N | N | N | N | N | N | N |
| A0A453ILR0 | N | N | N | N | N | N | N |
| A0A453IMG0 | N | N | N | N | N | N | N |
| A0A453PID0 | N | N | N | N | N | N | N |
| A0A453PIF1 | N | N | N | N | N | N | N |
| A0A453R9L3 | N | N | N | N | N | N | N |
| A0A453RA19 | N | N | N | N | N | N | N |
| A0A484KJP2 | N | N | N | N | N | N | N |
| A0A4Y7JLN2 | N | N | N | N | N | N | N |
| A0A699I1B2 | N | N | N | N | N | N | N |
| A0A699YWV8 | N | N | N | N | N | N | N |
| A0A6A4PUD9 | N | N | N | N | N | N | N |
| A0A6A4PV11 | N | N | N | N | N | N | N |
| A0A7J6V6B3 | N | N | N | N | N | N | N |
| A0A7J8YFK8 | N | N | N | N | N | N | N |
| A0A7J9LDI1 | N | N | N | N | N | N | N |
| A0A7J9MJ22 | N | N | N | N | N | N | N |
| A0A803Q4I0 | N | N | N | N | N | N | N |
| A0A835K942 | N | N | N | N | N | N | N |
| A0A835VW51 | N | N | N | N | N | N | N |
| A0A843VS65 | N | N | N | N | N | N | N |
| A0A8J5ERB3 | N | N | N | N | N | N | N |
| F2EI95 | N | N | N | N | N | N | N |
| M1CVR1 | N | N | N | N | N | N | N |
| M4CFD8 | N | N | N | N | N | N | N |
| W9SUD1 | N | N | N | N | N | N | N |
| A0A453CEY1 | N | N | N | N | N | N | N |

| | | | | | | | |
|---|---|---|---|---|---|---|---|
| A0A453CJ16 | N | N | N | N | N | N | N |
| A0A453RA61 | N | N | N | N | N | N | N |
| A0A453FGC5 | N | N | N | N | N | N | N |
| A0A0E0LA24 | N | N | N | N | N | N | N |
| A0A2N9EVX4 | N | N | N | N | N | N | N |
| A0A540LLF2 | N | N | N | N | N | N | N |
| A0A5N5K5I2 | N | N | N | N | N | N | N |
| A0A061EZT6 | N | N | N | N | N | N | N |
| A0A7J8RMD8 | N | N | N | N | N | N | N |
| A0A811Q0I4 | N | N | N | N | N | N | N |
| A0A022PRZ8 | N | N | N | N | N | N | N |
| I1P3Z9 | N | N | N | N | N | N | N |
| A0A103Y0P6 | N | N | N | N | N | N | N |
| Q0DTV5 | N | N | N | N | N | N | N |
| A0A0D9YXW9 | N | N | N | N | N | N | N |
| M8A8N7 | N | N | N | N | N | N | N |
| A0A6P5YSM4 | N | N | N | N | N | N | N |
| A0A0D3GEZ2 | N | N | N | N | N | N | N |
| A0A061FBX3 | N | N | N | N | N | N | N |
| A0A368QTB9 | N | N | N | N | N | N | N |
| A0A3Q7FVV8 | N | N | N | N | N | N | N |
| A0A4S4E8Z8 | N | N | N | N | N | N | N |
| A0A5J5ADD1 | N | N | N | N | N | N | N |
| A0A2P5BG78 | N | N | N | N | N | N | N |
| A0A328DTB9 | N | N | N | N | N | N | N |
| A0A7J8N7B8 | N | N | N | N | N | N | N |
| A0A1D6Q9V3 | N | N | N | NC | N | N | N |
| A0A834YU87 | NC | CN | N | CN | NC | N | N |
| A0A3L6DDM5 | NC | CN | N | CN | NC | N | N |
| A0A5K0YSB8 | NC | CN | N | CN | NC | N | N |
| A0A7I8KVN7 | N | N | N | CN | NC | N | N |
| A0A3B6TP41 | N | N | N | CN | NC | N | N |
| A0A453RA54 | N | N | N | CN | NC | N | N |
| A0A8I6YDC6 | N | N | N | CN | NC | N | N |
| M8BIV9 | N | N | N | CN | NC | N | N |
| A0A1S2XM79 | N | N | N | CN | NC | N | N |
| A0A2I0B0W0 | NC | N | N | CN | NC | N | N |
| A0A7I8J427 | NC | N | N | CN | NC | N | N |
| A0A7I8KUE5 | NC | N | N | CN | NC | N | N |
| A0A3Q7Y8H3 | NCN | N | N | CN | NC | N | N |
| A0A6A5NXI6 | NCN | N | N | CN | NC | N | N |
| A0A3Q7XMA7 | CNC | N | N | CNC | NC | N | N |
| A0A3Q7YCD9 | N | N | N | CNC | NC | N | N |
| A0A1D6HJI5 | NC | N | N | CNC | NC | N | N |
| A0A0D3FA61 | NC | NC | N | CNC | NC | N | N |
| A0A0E0NKM5 | NC | NC | N | CNC | NC | N | N |
| K7LDH0 | N | CN | N | N | NC | N | N |

| | | | | | | | |
|---|---|---|---|---|---|---|---|
| A0A834ZZ79 | N | CN | N | N | NC | N | N |
| A0A0E0NKM3 | N | CN | N | N | NC | N | N |
| A0A1S3XKK6 | N | CN | N | N | NC | N | N |
| A0A4Y7ILA6 | N | N | N | N | NC | N | N |
| A0A368QT28 | N | N | N | N | NC | N | N |
| A0A0D9YXW8 | N | N | N | N | NC | N | N |
| A0A0E0GCT4 | N | N | N | N | NC | N | N |
| A0A0V0H8I8 | N | N | N | N | NC | N | N |
| A0A199VRI9 | N | N | N | N | NC | N | N |
| A0A1D6NR01 | N | N | N | N | NC | N | N |
| A0A1E5UZ10 | N | N | N | N | NC | N | N |
| A0A1S3ZWA9 | N | N | N | N | NC | N | N |
| A0A1S4CQA4 | N | N | N | N | NC | N | N |
| A0A1U7Y1T4 | N | N | N | N | NC | N | N |
| A0A1U8E157 | N | N | N | N | NC | N | N |
| A0A1U8E2D8 | N | N | N | N | NC | N | N |
| A0A1U8HFF6 | N | N | N | N | NC | N | N |
| A0A1U8HLH0 | N | N | N | N | NC | N | N |
| A0A2G2WTB7 | N | N | N | N | NC | N | N |
| A0A2G2XSK6 | N | N | N | N | NC | N | N |
| A0A2G2YWA4 | N | N | N | N | NC | N | N |
| A0A2G2ZIZ4 | N | N | N | N | NC | N | N |
| A0A2G3BRG9 | N | N | N | N | NC | N | N |
| A0A2G3CHA9 | N | N | N | N | NC | N | N |
| A0A2T7E1A0 | N | N | N | N | NC | N | N |
| A0A3B6TH27 | N | N | N | N | NC | N | N |
| A0A3L6PDW3 | N | N | N | N | NC | N | N |
| A0A3Q7E794 | N | N | N | N | NC | N | N |
| A0A3Q7FV91 | N | N | N | N | NC | N | N |
| A0A4U6WF80 | N | N | N | N | NC | N | N |
| A0A4V3WK47 | N | N | N | N | NC | N | N |
| A0A6N2BW27 | N | N | N | N | NC | N | N |
| A0A6N2C6V0 | N | N | N | N | NC | N | N |
| A0A6P5F8U5 | N | N | N | N | NC | N | N |
| A0A7N0TFZ3 | N | N | N | N | NC | N | N |
| A0A804QZ81 | N | N | N | N | NC | N | N |
| A0A804UIG9 | N | N | N | N | NC | N | N |
| A0A811PE78 | N | N | N | N | NC | N | N |
| A0A811Q107 | N | N | N | N | NC | N | N |
| A0A833R268 | N | N | N | N | NC | N | N |
| A0A835DS02 | N | N | N | N | NC | N | N |
| A0A835F662 | N | N | N | N | NC | N | N |
| A0A835FSY0 | N | N | N | N | NC | N | N |
| A0A8B8ZQL0 | N | N | N | N | NC | N | N |
| A0A8J5SHH7 | N | N | N | N | NC | N | N |
| B8AI26 | N | N | N | N | NC | N | N |
| B9F2K6 | N | N | N | N | NC | N | N |

| | | | | | | | |
|---|---|---|---|---|---|---|---|
| D8U9T4 | N | N | N | N | NC | N | N |
| M0ZSZ7 | N | N | N | N | NC | N | N |
| M1AL41 | N | N | N | N | NC | N | N |
| M1AL42 | N | N | N | N | NC | N | N |
| M1CJJ0 | N | N | N | N | NC | N | N |
| A0A5P1EJQ4 | N | N | N | N | NC | N | N |
| A0A4S4EGN0 | N | N | N | N | NC | N | N |
| A0A1B5GE61 | N | N | N | N | NC | N | N |
| A0A1B5GEN6 | N | N | N | N | NC | N | N |
| A0A3B6RGE0 | N | N | N | N | NC | N | N |
| A0A3B6SGB2 | N | N | N | N | NC | N | N |
| A0A4U6VA55 | N | N | N | N | NC | N | N |
| A0A6V7P7E1 | N | N | N | N | NC | N | N |
| A0A816M7G6 | N | N | N | N | NC | N | N |
| A0A816YI10 | N | N | N | N | NC | N | N |
| A0A8D9HLF7 | N | N | N | N | NC | N | N |
| A0A0E0CR44 | N | N | N | N | NC | N | N |
| A0A0P0VP64 | N | N | N | N | NC | N | N |
| A0A445C7Q4 | N | N | N | N | NC | N | N |
| A0A453PIC2 | N | N | N | N | NC | N | N |
| A0A453PID6 | N | N | N | N | NC | N | N |
| A0A7N0TFY0 | N | N | N | N | NC | N | N |
| A0A8B9AQF8 | N | N | N | N | NC | N | N |
| M8CGP1 | N | N | N | N | NC | N | N |
| Q0DD57 | N | N | N | N | NC | N | N |
| A0A6I9QYN5 | N | N | N | N | NC | N | N |
| A0A7N0TGQ7 | N | N | N | N | NC | N | N |
| A0A7N0UBA2 | N | N | N | N | NC | N | N |
| A0A8B8ZZ11 | N | N | N | N | NC | N | N |
| A0A453PIE6 | N | N | N | N | NC | N | N |
| A0A3L6RUG3 | N | N | N | N | NC | N | N |
| A0A3L6RVD2 | N | N | N | N | NC | N | N |
| A0A453PID5 | N | N | N | N | NC | N | N |
| A0A6I9QHI1 | N | N | N | N | NC | N | N |
| A0A8B7BGA2 | N | N | N | N | NC | N | N |
| F2CWD4 | N | N | N | N | NC | N | N |
| R7W3Q5 | N | N | N | N | NC | N | N |
| I1P3Z8 | N | N | N | N | NC | N | N |
| A0A7J0D8K0 | N | N | N | N | NC | N | N |
| A0A4Y7JNV2 | N | N | N | N | NC | N | N |
| Q6Z2H9 | N | N | N | N | NC | N | N |
| A0A1U8I2W3 | N | N | N | N | NC | N | N |
| A0A8J5E8I9 | N | N | N | N | NC | N | N |
| A0A6I9S363 | N | N | N | N | NC | N | N |
| J3LGT9 | N | N | N | N | NC | N | N |
| A0A7N0TGW6 | N | N | N | N | NC | N | N |
| M8AWV1 | N | N | N | N | NC | N | N |

| | | | | | | | |
|---|---|---|---|---|---|---|---|
| A0A2U1L4B8 | NC | N | N | N | NC | N | N |
| A0A5N6QS39 | NC | N | N | N | NC | N | N |
| Q9CAB8 | NC | N | N | N | NC | N | N |
| A0A0A9HY31 | NC | N | N | N | NC | N | N |
| A0A328DTW7 | NC | N | N | N | NC | N | N |
| A0A3B6RC97 | NC | N | N | N | NC | N | N |
| A0A3B6RGA0 | NC | N | N | N | NC | N | N |
| A0A484LYT1 | NC | N | N | N | NC | N | N |
| A0A6N2BKX4 | NC | N | N | N | NC | N | N |
| A0A8I6Y706 | NC | N | N | N | NC | N | N |
| A0A7G2DYG9 | NC | N | N | N | NC | N | N |
| Q0DXT5 | NC | N | N | N | NC | N | N |
| A0A8J5BTA9 | NC | N | N | N | NC | N | N |
| A0A453R9K8 | NC | N | N | N | NC | N | N |
| A0A2G5C744 | NC | N | N | N | NC | N | N |
| A0A835F680 | N | N | N | NC | NC | N | N |
| A0A6V7P7G0 | N | N | N | NC | NC | N | N |
| Q9CAB7 | NC | N | N | NC | NC | N | N |
| A0A068V705 | NC | N | N | NC | NC | N | N |
| V7C517 | NC | N | N | NC | NC | N | N |
| A0A6A6LX87 | NC | N | N | NC | NC | N | N |
| A0A804U949 | NC | NCN | N | NC | NC | N | N |
| A0A8B8ZT23 | N | N | N | CN | NCN | N | N |
| A0A2I0B4L0 | NCN | N | N | CNC | NCN | N | N |
| A0A811S7Q3 | N | N | N | N | NCN | N | N |
| A0A4U6UVZ7 | N | N | N | N | NCN | N | N |
| K3XZD9 | N | N | N | N | NCN | N | N |
| A0A0D9VKP5 | N | N | N | N | NCN | N | N |
| A0A0L9UTA8 | N | N | N | N | NCN | N | N |
| A0A6L2K0K4 | NCN | N | N | N | NCN | N | N |
| A0A6L2KCA0 | NCN | N | N | N | NCN | N | N |
| A0A8J4BFE3 | N | CN | NC | CN | CN | CN | NC |
| A0A2K3MB38 | NC | CN | NC | CN | CN | CN | NC |
| V4S036 | CNC | CNC | NC | CNC | CNC | CN | NC |
| A0A834ZY80 | CN | N | CNC | N | N | CN | NC |
| A0A3L6S149 | N | N | NC | N | N | CN | NC |
| A0A0E0PVX8 | N | N | NCN | N | N | CN | NC |
| A0A0E0PVX9 | N | N | NCN | N | N | CN | NC |
| I1H894 | N | NC | CNC | N | NC | CN | NC |
| A0A843XNC8 | N | NC | NC | N | NC | CN | NC |
| A0A5J9UXN3 | N | CNC | CNC | N | CN | CNC | NC |
| A0A368QTG1 | N | CNC | CNC | N | CN | CNC | NC |
| A0A3B6TN25 | N | CNC | CNC | N | CN | CNC | NC |
| A0A836B6K4 | N | NC | NC | N | CN | CNC | NC |
| A0A2K3E6M1 | N | NC | NC | N | CN | CNC | NC |
| A0A1Y1ILK1 | N | NC | NC | N | CN | CNC | NC |
| A0A2S3HKL3 | CN | CNC | CNC | CN | CNC | CNC | NC |

| | | | | | | | |
|---|---|---|---|---|---|---|---|
| A0A317YCV2 | CN | CNC | NC | CN | CNC | CNC | NC |
| A0A0R0F101 | CN | CNC | NC | CN | CNC | CNC | NC |
| A0A445FL87 | CN | CNC | NC | CN | CNC | CNC | NC |
| A0A0P0UNR5 | N | CNC | NC | CN | CNC | CNC | NC |
| A0A2P6TLZ1 | N | CNC | NC | CN | CNC | CNC | NC |
| A0A2P6VI04 | N | CNC | NC | CN | CNC | CNC | NC |
| D8UI18 | N | CNC | NC | CN | CNC | CNC | NC |
| I0YMZ3 | N | CNC | NC | CN | CNC | CNC | NC |
| A0A0D2MXJ9 | N | CNC | NC | CN | CNC | CNC | NC |
| A0A2V0P0J4 | N | CNC | NC | CN | CNC | CNC | NC |
| I0YMZ2 | N | CNC | NC | CN | CNC | CNC | NC |
| A0A445F7T5 | NC | CNC | NC | CN | CNC | CNC | NC |
| A0A6V7QLT2 | NC | CNC | NC | CN | CNC | CNC | NC |
| A0A445F7X2 | NCN | CNC | NC | CN | CNC | CNC | NC |
| K3XYR9 | CNC | CNC | CNC | CNC | CNC | CNC | NC |
| A0A0E0A7R8 | CNC | CNC | CNC | CNC | CNC | CNC | NC |
| A0A0E0HNL0 | CNC | CNC | CNC | CNC | CNC | CNC | NC |
| A0A0E0PVY2 | CNC | CNC | CNC | CNC | CNC | CNC | NC |
| A3BAC7 | CNC | CNC | CNC | CNC | CNC | CNC | NC |
| I1Q1A0 | CNC | CNC | CNC | CNC | CNC | CNC | NC |
| F2CS41 | N | CNC | CNC | CNC | CNC | CNC | NC |
| F2CW46 | N | CNC | CNC | CNC | CNC | CNC | NC |
| A0A0D9WNY1 | NC | CNC | CNC | CNC | CNC | CNC | NC |
| A0A2V0NK14 | CN | CNC | NC | CNC | CNC | CNC | NC |
| A0A7J7D7A7 | CNC | CNC | NC | CNC | CNC | CNC | NC |
| A0A1U8AXB3 | CNC | CNC | NC | CNC | CNC | CNC | NC |
| G1AUC6 | CNC | CNC | NC | CNC | CNC | CNC | NC |
| A0A6A5NNE6 | NC | CNC | NC | CNC | CNC | CNC | NC |
| A0A2T7E1A9 | CN | CNC | CNC | N | CNC | CNC | NC |
| J3MD01 | N | NC | CNC | N | CNC | CNC | NC |
| A0A811SAH7 | N | NC | CNC | N | CNC | CNC | NC |
| M7ZN37 | NC | NC | CNC | N | CNC | CNC | NC |
| A0A6A2Z397 | NC | CNC | NC | N | CNC | CNC | NC |
| A0A8J4LWU3 | N | NC | NC | N | CNC | CNC | NC |
| A0A1D1ZQY2 | N | NC | NC | N | CNC | CNC | NC |
| A0A3L6RXN9 | N | NC | NC | N | CNC | CNC | NC |
| A0A835YNP5 | N | NC | NC | N | CNC | CNC | NC |
| A0A5J9TMB1 | N | NC | NC | N | CNC | CNC | NC |
| A0A383V5U8 | N | NC | NC | N | CNC | CNC | NC |
| A0A8J4FKW0 | N | NC | NC | N | CNC | CNC | NC |
| A0A6A2WK72 | NC | NC | NC | N | CNC | CNC | NC |
| A0A0E0PVX3 | N | CNC | CNC | CN | N | CNC | NC |
| A0A453FG79 | N | CNC | CNC | CN | N | CNC | NC |
| A0A8J8YQR4 | N | NC | CNC | CN | N | CNC | NC |
| A0A804LQL7 | N | N | NCN | CN | N | CNC | NC |
| A0A835WCG4 | N | N | CN | N | N | CNC | NC |
| A0A3L6RYE3 | CNC | NC | CNC | N | N | CNC | NC |

| | | | | | | | |
|---|---|---|---|---|---|---|---|
| A0A3B6FP68 | N | NC | CNC | N | N | CNC | NC |
| A0A3B6UAI5 | N | NC | CNC | N | N | CNC | NC |
| Q9M4D5 | N | NC | CNC | N | N | CNC | NC |
| X4XVL7 | N | NC | CNC | N | N | CNC | NC |
| X4Y4U8 | N | NC | CNC | N | N | CNC | NC |
| A0A0E0A7R5 | N | NC | CNC | N | N | CNC | NC |
| A0A1D6HJH6 | N | NC | CNC | N | N | CNC | NC |
| A0A2T7FBU8 | N | NC | CNC | N | N | CNC | NC |
| A0A3B6GVV5 | N | NC | CNC | N | N | CNC | NC |
| A0A6G1F6K4 | N | NC | CNC | N | N | CNC | NC |
| B6TKU1 | N | NC | CNC | N | N | CNC | NC |
| B6UDK3 | N | NC | CNC | N | N | CNC | NC |
| M7ZYL2 | N | NC | CNC | N | N | CNC | NC |
| M8BSL3 | N | NC | CNC | N | N | CNC | NC |
| C5XZA4 | N | NC | CNC | N | N | CNC | NC |
| A0A096UTL6 | N | NC | CNC | N | N | CNC | NC |
| A0A0D9WNX8 | N | NC | CNC | N | N | CNC | NC |
| A0A1D6HJI0 | N | NC | CNC | N | N | CNC | NC |
| A0A1E5UUD9 | N | NC | CNC | N | N | CNC | NC |
| A0A2S3HKL9 | N | NC | CNC | N | N | CNC | NC |
| A0A3B6PRL7 | N | NC | CNC | N | N | CNC | NC |
| A0A3B6QHJ0 | N | NC | CNC | N | N | CNC | NC |
| A0A3B6RCS5 | N | NC | CNC | N | N | CNC | NC |
| A0A3L6PCZ9 | N | NC | CNC | N | N | CNC | NC |
| A0A3L6QD45 | N | NC | CNC | N | N | CNC | NC |
| A0A453PIB2 | N | NC | CNC | N | N | CNC | NC |
| A0A5J9TK66 | N | NC | CNC | N | N | CNC | NC |
| A0A5J9UZC0 | N | NC | CNC | N | N | CNC | NC |
| B6TIA8 | N | NC | CNC | N | N | CNC | NC |
| K3YZS5 | N | NC | CNC | N | N | CNC | NC |
| M7YU43 | N | NC | CNC | N | N | CNC | NC |
| M8A9K0 | N | NC | CNC | N | N | CNC | NC |
| X4Y244 | N | NC | CNC | N | N | CNC | NC |
| A0A0N7KG19 | N | NC | CNC | N | N | CNC | NC |
| I1GZ91 | N | NC | CNC | N | N | CNC | NC |
| I1H890 | N | NC | CNC | N | N | CNC | NC |
| A0A1B5GE54 | N | NC | CNC | N | N | CNC | NC |
| A0A2T7E1B3 | N | NC | CNC | N | N | CNC | NC |
| A0A2T8JE58 | N | NC | CNC | N | N | CNC | NC |
| I1IEG7 | N | NC | CNC | N | N | CNC | NC |
| A0A1D6HJH9 | N | NC | NC | N | N | CNC | NC |
| A0A3L6PDN3 | N | NC | NC | N | N | CNC | NC |
| A0A443N934 | N | NC | NC | N | N | CNC | NC |
| C5Z7Z5 | N | NC | NC | N | N | CNC | NC |
| M8BSQ5 | N | NC | NC | N | N | CNC | NC |
| A0A1D6HJI2 | N | NC | NC | N | N | CNC | NC |
| A0A2T7E1A3 | N | NC | NC | N | N | CNC | NC |

| | | | | | | | |
|---|---|---|---|---|---|---|---|
| A0A804QYR4 | N | NC | NC | N | N | CNC | NC |
| A0A834ZVU5 | N | NC | NC | N | N | CNC | NC |
| A0A3L6DE89 | N | NC | NC | N | N | CNC | NC |
| B6T0R9 | N | NC | NC | N | N | CNC | NC |
| C0P2H4 | N | NC | NC | N | N | CNC | NC |
| C5Z7Z4 | N | NC | NC | N | N | CNC | NC |
| M7ZHE9 | N | NC | NC | N | N | CNC | NC |
| A0A3L6S5C7 | N | N | NCN | N | N | CNC | NC |
| A3BAC6 | N | N | NCN | N | N | CNC | NC |
| A0A5J9TKB4 | N | N | NCN | N | N | CNC | NC |
| A0A811RIY1 | N | N | NCN | N | N | CNC | NC |
| A0A0E0A7R4 | N | N | NCN | N | N | CNC | NC |
| Q652U8 | N | N | NCN | N | N | CNC | NC |
| A0A0D9WNY0 | N | N | NCN | N | N | CNC | NC |
| A0A0E0PVX4 | N | N | NCN | N | N | CNC | NC |
| A0A0E0PVX5 | N | N | NCN | N | N | CNC | NC |
| A0A0E0PVX7 | N | N | NCN | N | N | CNC | NC |
| A0A5J9TMC2 | N | N | NCN | N | N | CNC | NC |
| A2YBC8 | N | N | NCN | N | N | CNC | NC |
| I1Q199 | N | N | NCN | N | N | CNC | NC |
| Q656N6 | N | N | NCN | N | N | CNC | NC |
| A0A3B6PIG2 | N | N | NCN | N | N | CNC | NC |
| A0A453NKL4 | N | N | NCN | N | N | CNC | NC |
| E7D433 | N | N | NCN | N | N | CNC | NC |
| A0A3B6SBR4 | N | N | NCN | N | N | CNC | NC |
| A0A453PIE0 | N | CNC | CNC | CNC | NC | CNC | NC |
| A0A834ZVV3 | NC | CNC | CNC | CNC | NC | CNC | NC |
| Q6Z2H7 | NCN | CNC | CNC | CNC | NC | CNC | NC |
| A0A0E0CR46 | NCN | CNC | CNC | CNC | NC | CNC | NC |
| A0A0E0NKM4 | NCN | CNC | CNC | CNC | NC | CNC | NC |
| A0A0E0K4Z2 | N | NC | CNC | CNC | NC | CNC | NC |
| A0A0E0ISE1 | NC | NC | CNC | CNC | NC | CNC | NC |
| A0A7J6HLA5 | NC | NC | NC | CNC | NC | CNC | NC |
| A0A3Q7XVA8 | NC | NC | NC | CNC | NC | CNC | NC |
| A0A6A4PV96 | NC | NC | NC | CNC | NC | CNC | NC |
| A0A072VEZ4 | NC | NC | NC | CNC | NC | CNC | NC |
| A0A7J7M7I7 | NC | NC | NC | CNC | NC | CNC | NC |
| A0A5B6ZQP1 | NCN | NC | NC | CNC | NC | CNC | NC |
| J3LGU1 | N | NC | CNC | N | NC | CNC | NC |
| A0A3L6E9U6 | N | NC | CNC | N | NC | CNC | NC |
| A0A3L6QF18 | N | NC | CNC | N | NC | CNC | NC |
| A0A8B9AFX5 | N | NC | CNC | N | NC | CNC | NC |
| A0A8B9AG44 | N | NC | CNC | N | NC | CNC | NC |
| A0A2S3GT06 | N | NC | CNC | N | NC | CNC | NC |
| A0A368PRH4 | N | NC | CNC | N | NC | CNC | NC |
| A0A4U6WFJ4 | N | NC | CNC | N | NC | CNC | NC |
| A0A8J5S331 | N | NC | CNC | N | NC | CNC | NC |

| | | | | | | | |
|---|---|---|---|---|---|---|---|
| M8AVG3 | N | NC | CNC | N | NC | CNC | NC |
| C5XZA3 | N | NC | CNC | N | NC | CNC | NC |
| C5Z7Z3 | N | NC | CNC | N | NC | CNC | NC |
| A0A0A9VHC2 | N | NC | CNC | N | NC | CNC | NC |
| A0A3B6NTG7 | N | NC | CNC | N | NC | CNC | NC |
| A0A4U6WJ71 | N | NC | CNC | N | NC | CNC | NC |
| A0A8I6Y459 | N | NC | CNC | N | NC | CNC | NC |
| A2YCT4 | N | NC | CNC | N | NC | CNC | NC |
| A3AB40 | N | NC | CNC | N | NC | CNC | NC |
| B6SPX6 | N | NC | CNC | N | NC | CNC | NC |
| I1P401 | N | NC | CNC | N | NC | CNC | NC |
| A0A3S3M0W7 | N | NC | CNC | N | NC | CNC | NC |
| A0A1E5UUI9 | N | NC | CNC | N | NC | CNC | NC |
| C4J0B8 | N | NC | CNC | N | NC | CNC | NC |
| A0A2K2D5E4 | N | NC | CNC | N | NC | CNC | NC |
| A0A6G1F6K7 | N | NC | CNC | N | NC | CNC | NC |
| A0A835FU41 | NC | NC | CNC | N | NC | CNC | NC |
| A0A804PNA1 | NC | NC | CNC | N | NC | CNC | NC |
| B6T641 | NC | NC | CNC | N | NC | CNC | NC |
| C0PBJ1 | NC | NC | CNC | N | NC | CNC | NC |
| A0A6I9QYN4 | N | NC | NC | N | NC | CNC | NC |
| A0A2C9U662 | N | NC | NC | N | NC | CNC | NC |
| A0A6A4PV04 | N | NC | NC | N | NC | CNC | NC |
| A0A6A4PVL3 | N | NC | NC | N | NC | CNC | NC |
| A0A8J5C4X9 | N | NC | NC | N | NC | CNC | NC |
| Q9XFY1 | N | NC | NC | N | NC | CNC | NC |
| A0A087SUC1 | N | NC | NC | N | NC | CNC | NC |
| A0A2C9U706 | N | NC | NC | N | NC | CNC | NC |
| A0A7J7DUS2 | NC | NC | NC | N | NC | CNC | NC |
| A0A2C9U609 | NC | NC | NC | N | NC | CNC | NC |
| A0A2C9U643 | NCN | NC | NC | N | NC | CNC | NC |
| A0A3B6TP37 | N | NC | NCN | N | NC | CNC | NC |
| A0A8J6BUQ8 | NC | NC | NC | NCN | NC | CNC | NC |
| A0A1E5VNQ5 | NCN | NC | NC | NCN | NC | CNC | NC |
| A0A1S3VM95 | NCN | NC | NC | NCN | NC | CNC | NC |
| A0A2S3GT01 | N | NC | CNC | N | NCN | CNC | NC |
| A0A2T7FBT7 | N | NC | CNC | N | NCN | CNC | NC |
| A0A0E0K4Z0 | N | NC | CNC | N | NCN | CNC | NC |
| A0A3L6PF33 | N | NC | CNC | N | NCN | CNC | NC |
| B6TWY2 | NC | NC | CNC | N | NCN | CNC | NC |
| A0A811SF91 | N | NC | NC | N | CN | NC | NC |
| A0A6A4L8J3 | CN | NC | NC | CN | CNC | NC | NC |
| F4I4P8 | CNC | NC | NC | CN | CNC | NC | NC |
| A0A822YJ97 | NC | NC | NC | CN | CNC | NC | NC |
| A0A816WVX7 | CNC | NC | NC | CNC | CNC | NC | NC |
| A0A835L9I8 | CNC | NC | NC | CNC | CNC | NC | NC |
| A0A3P6HBC7 | NC | NC | NC | CNC | CNC | NC | NC |

| | | | | | | | |
|---|---|---|---|---|---|---|---|
| A0A453R9K2 | N | NC | NC | N | CNC | NC | NC |
| A0A453ILR2 | N | NC | NC | N | CNC | NC | NC |
| A0A2P5C370 | NC | NC | NC | N | CNC | NC | NC |
| A0A7C8ZMH0 | NCN | NC | NC | NCN | CNC | NC | NC |
| A0A2K3KZW5 | N | NC | NC | CN | N | NC | NC |
| A3AB38 | N | NC | NC | N | N | NC | NC |
| Q6Z2H8 | N | NC | NC | N | N | NC | NC |
| A0A3B6JLI6 | N | NC | NC | N | N | NC | NC |
| A0A811S7Y7 | N | NC | NC | N | N | NC | NC |
| A0A2K2DPB0 | N | NC | NC | N | N | NC | NC |
| A0A0E0CR45 | N | N | NCN | N | N | NC | NC |
| A0A699L8B9 | NC | NC | NC | NC | N | NC | NC |
| A0A1P8ANL6 | CN | NC | NC | CN | NC | NC | NC |
| A0A1P8ATG9 | CNC | NC | NC | CN | NC | NC | NC |
| A0A5S9VQ31 | N | NC | NC | CN | NC | NC | NC |
| A0A654EDX8 | N | NC | NC | CN | NC | NC | NC |
| A0A1S3E6E6 | N | NC | NC | CN | NC | NC | NC |
| A0A6P4B0V8 | N | NC | NC | CN | NC | NC | NC |
| A0A7J6EAH1 | N | NC | NC | CN | NC | NC | NC |
| A0A4P1RP83 | N | NC | NC | CN | NC | NC | NC |
| A0A2P5ABC8 | N | NC | NC | CN | NC | NC | NC |
| A0A161WQ46 | N | NC | NC | CN | NC | NC | NC |
| I3SLW8 | NC | NC | NC | CN | NC | NC | NC |
| C6TFE2 | NC | NC | NC | CN | NC | NC | NC |
| A0A022QS55 | NC | NC | NC | CN | NC | NC | NC |
| A0A0B2P2J5 | NC | NC | NC | CN | NC | NC | NC |
| A0A0B2S9L2 | NC | NC | NC | CN | NC | NC | NC |
| A0A0S3T1J1 | NC | NC | NC | CN | NC | NC | NC |
| A0A151R4M2 | NC | NC | NC | CN | NC | NC | NC |
| A0A175YHF7 | NC | NC | NC | CN | NC | NC | NC |
| A0A1S3XH22 | NC | NC | NC | CN | NC | NC | NC |
| A0A218WX96 | NC | NC | NC | CN | NC | NC | NC |
| A0A2I0IX97 | NC | NC | NC | CN | NC | NC | NC |
| A0A2N9FBL5 | NC | NC | NC | CN | NC | NC | NC |
| A0A394DGP7 | NC | NC | NC | CN | NC | NC | NC |
| A0A444XZB8 | NC | NC | NC | CN | NC | NC | NC |
| A0A444YAY1 | NC | NC | NC | CN | NC | NC | NC |
| A0A4D6LMY8 | NC | NC | NC | CN | NC | NC | NC |
| A0A5A7PBM0 | NC | NC | NC | CN | NC | NC | NC |
| A0A6A5NYF6 | NC | NC | NC | CN | NC | NC | NC |
| A0A6C0QI75 | NC | NC | NC | CN | NC | NC | NC |
| A0A6C0QJX9 | NC | NC | NC | CN | NC | NC | NC |
| A0A7C8ZKH2 | NC | NC | NC | CN | NC | NC | NC |
| A0A803M905 | NC | NC | NC | CN | NC | NC | NC |
| A0A8B8M1R1 | NC | NC | NC | CN | NC | NC | NC |
| A0A8B8M2V4 | NC | NC | NC | CN | NC | NC | NC |
| B7FI43 | NC | NC | NC | CN | NC | NC | NC |

| | | | | | | | |
|---|---|---|---|---|---|---|---|
| I3SNU8 | NC | NC | NC | CN | NC | NC | NC |
| V7C8N0 | NC | NC | NC | CN | NC | NC | NC |
| A0A8D9M9T8 | NC | NC | NC | CN | NC | NC | NC |
| A0A087HH83 | NC | NC | NC | CN | NC | NC | NC |
| A0A0A0KV83 | NC | NC | NC | CN | NC | NC | NC |
| A0A0D2S4X4 | NC | NC | NC | CN | NC | NC | NC |
| A0A1S3VS60 | NC | NC | NC | CN | NC | NC | NC |
| A0A1U8K1N6 | NC | NC | NC | CN | NC | NC | NC |
| A0A3S3R898 | NC | NC | NC | CN | NC | NC | NC |
| A0A438CA69 | NC | NC | NC | CN | NC | NC | NC |
| A0A4S8IIM0 | NC | NC | NC | CN | NC | NC | NC |
| A0A5D2D0M6 | NC | NC | NC | CN | NC | NC | NC |
| A0A5D2GZX0 | NC | NC | NC | CN | NC | NC | NC |
| A0A5D2LH05 | NC | NC | NC | CN | NC | NC | NC |
| A0A5D2R3Y2 | NC | NC | NC | CN | NC | NC | NC |
| A0A5D2VHP1 | NC | NC | NC | CN | NC | NC | NC |
| A0A5D2ZQY3 | NC | NC | NC | CN | NC | NC | NC |
| A0A5J5S1F8 | NC | NC | NC | CN | NC | NC | NC |
| A0A5J5W8S0 | NC | NC | NC | CN | NC | NC | NC |
| A0A6P4NML5 | NC | NC | NC | CN | NC | NC | NC |
| A0A6P5XDT3 | NC | NC | NC | CN | NC | NC | NC |
| A0A6P5XF44 | NC | NC | NC | CN | NC | NC | NC |
| A0A803MFF2 | NC | NC | NC | CN | NC | NC | NC |
| A0A804KPF6 | NC | NC | NC | CN | NC | NC | NC |
| A0A8J5ZE30 | NC | NC | NC | CN | NC | NC | NC |
| Q70DJ9 | NC | NC | NC | CN | NC | NC | NC |
| A0A0K9PDD4 | NC | NC | NC | CN | NC | NC | NC |
| A0A2P2LMG5 | NC | NC | NC | CN | NC | NC | NC |
| A0A2P2LMI0 | NC | NC | NC | CN | NC | NC | NC |
| A0A371F2Q6 | NC | NC | NC | CN | NC | NC | NC |
| A0A394DFI0 | NC | NC | NC | CN | NC | NC | NC |
| A0A4Y7IKS5 | NC | NC | NC | CN | NC | NC | NC |
| A0A5B6V2Q6 | NC | NC | NC | CN | NC | NC | NC |
| A0A6P4BW17 | NC | NC | NC | CN | NC | NC | NC |
| I3SSS0 | NC | NC | NC | CN | NC | NC | NC |
| K7LDH4 | NC | NC | NC | CN | NC | NC | NC |
| A0A426X3D5 | NC | NC | NC | CN | NC | NC | NC |
| A0A6P4B6B9 | NC | NC | NC | CN | NC | NC | NC |
| A0A0V0GNT7 | NC | NC | NC | CN | NC | NC | NC |
| A0A816ZI42 | NC | NC | NC | CN | NC | NC | NC |
| W1NF78 | NC | NC | NC | CN | NC | NC | NC |
| A0A833R9H7 | NC | NC | NC | CN | NC | NC | NC |
| A0A834LBH2 | NC | NC | NC | CNC | NC | NC | NC |
| A0A5B6ZTQ6 | NC | NC | NC | CNC | NC | NC | NC |
| A0A0R0I7H1 | N | NC | NC | N | NC | NC | NC |
| A0A2P2LMH1 | N | NC | NC | N | NC | NC | NC |
| A0A078FHM7 | N | NC | NC | N | NC | NC | NC |

| | | | | | | | |
|---|---|---|---|---|---|---|---|
| A0A078GKU4 | N | NC | NC | N | NC | NC | NC |
| A0A087HQL1 | N | NC | NC | N | NC | NC | NC |
| A0A1D1XMH4 | N | NC | NC | N | NC | NC | NC |
| A0A2P5DAJ6 | N | NC | NC | N | NC | NC | NC |
| A0A397YNT5 | N | NC | NC | N | NC | NC | NC |
| A0A3P6EPE3 | N | NC | NC | N | NC | NC | NC |
| M4D7A9 | N | NC | NC | N | NC | NC | NC |
| A0A0R0I7K9 | N | NC | NC | N | NC | NC | NC |
| Q652U4 | N | NC | NC | N | NC | NC | NC |
| A0A0A9QHJ1 | N | NC | NC | N | NC | NC | NC |
| A0A0B2Q5B0 | N | NC | NC | N | NC | NC | NC |
| A0A0B2QM74 | N | NC | NC | N | NC | NC | NC |
| A0A0E0LA26 | N | NC | NC | N | NC | NC | NC |
| A0A0R0IDL0 | N | NC | NC | N | NC | NC | NC |
| A0A3L6S049 | N | NC | NC | N | NC | NC | NC |
| A0A445J0A7 | N | NC | NC | N | NC | NC | NC |
| A0A4D6LLE7 | N | NC | NC | N | NC | NC | NC |
| A0A565ATN5 | N | NC | NC | N | NC | NC | NC |
| A0A6G1CWF1 | N | NC | NC | N | NC | NC | NC |
| A0A8J5C7X9 | N | NC | NC | N | NC | NC | NC |
| A0A8J5W3L5 | N | NC | NC | N | NC | NC | NC |
| C4J9K6 | N | NC | NC | N | NC | NC | NC |
| C6T0F9 | N | NC | NC | N | NC | NC | NC |
| Q6Q7Y1 | N | NC | NC | N | NC | NC | NC |
| A0A1W0VSC1 | N | NC | NC | N | NC | NC | NC |
| A0A0S3T1J2 | N | NC | NC | N | NC | NC | NC |
| A0A175YHP3 | N | NC | NC | N | NC | NC | NC |
| A0A2S3HKM1 | N | NC | NC | N | NC | NC | NC |
| A0A2T7E1A1 | N | NC | NC | N | NC | NC | NC |
| A0A4D6LMX8 | N | NC | NC | N | NC | NC | NC |
| A0A5N5GZV3 | N | NC | NC | N | NC | NC | NC |
| A0A5N5L5Z8 | N | NC | NC | N | NC | NC | NC |
| A0A6J0JM97 | N | NC | NC | N | NC | NC | NC |
| A0A6J0PGF1 | N | NC | NC | N | NC | NC | NC |
| A0A6N2M3M4 | N | NC | NC | N | NC | NC | NC |
| A0A811S3K0 | N | NC | NC | N | NC | NC | NC |
| A0A834Z3J6 | N | NC | NC | N | NC | NC | NC |
| A0A8J5WSN6 | N | NC | NC | N | NC | NC | NC |
| B4FLY6 | N | NC | NC | N | NC | NC | NC |
| B6TL27 | N | NC | NC | N | NC | NC | NC |
| K3Y137 | N | NC | NC | N | NC | NC | NC |
| V4MAE1 | N | NC | NC | N | NC | NC | NC |
| A0A0E0E021 | N | NC | NC | N | NC | NC | NC |
| A0A0E0LA28 | N | NC | NC | N | NC | NC | NC |
| A0A0S3T1B6 | N | NC | NC | N | NC | NC | NC |
| A0A1U8B3D7 | N | NC | NC | N | NC | NC | NC |
| A0A3N6TUI1 | N | NC | NC | N | NC | NC | NC |

| | | | | | | | |
|---|---|---|---|---|---|---|---|
| A0A6A5NYP5 | N | NC | NC | N | NC | NC | NC |
| A0A822YX65 | N | NC | NC | N | NC | NC | NC |
| C5Z7Z7 | N | NC | NC | N | NC | NC | NC |
| A0A3Q0EM23 | N | NC | NC | N | NC | NC | NC |
| A0A6A4PUH6 | N | NC | NC | N | NC | NC | NC |
| A0A6A5NY75 | N | NC | NC | N | NC | NC | NC |
| R0ICY2 | N | NC | NC | N | NC | NC | NC |
| A0A498IC56 | N | NC | NC | N | NC | NC | NC |
| A0A5D2WAK1 | N | NC | NC | N | NC | NC | NC |
| A0A453RA44 | N | NC | NC | N | NC | NC | NC |
| A0A087HH77 | NC | NC | NC | N | NC | NC | NC |
| A0A5N6RC14 | NC | NC | NC | N | NC | NC | NC |
| A0A2P5BG65 | NC | NC | NC | N | NC | NC | NC |
| A0A078GFS4 | NC | NC | NC | N | NC | NC | NC |
| A0A0A9UBH6 | NC | NC | NC | N | NC | NC | NC |
| A0A0A9V9E9 | NC | NC | NC | N | NC | NC | NC |
| A0A0V0HHF2 | NC | NC | NC | N | NC | NC | NC |
| A0A178WD81 | NC | NC | NC | N | NC | NC | NC |
| A0A1J6IPK9 | NC | NC | NC | N | NC | NC | NC |
| A0A1S3XHN0 | NC | NC | NC | N | NC | NC | NC |
| A0A1S3ZXQ1 | NC | NC | NC | N | NC | NC | NC |
| A0A1S4DLG4 | NC | NC | NC | N | NC | NC | NC |
| A0A1U7Y596 | NC | NC | NC | N | NC | NC | NC |
| A0A1U8DTJ8 | NC | NC | NC | N | NC | NC | NC |
| A0A2G2YW69 | NC | NC | NC | N | NC | NC | NC |
| A0A314L6S3 | NC | NC | NC | N | NC | NC | NC |
| A0A484KY92 | NC | NC | NC | N | NC | NC | NC |
| A0A565AZK5 | NC | NC | NC | N | NC | NC | NC |
| A0A5S9WR76 | NC | NC | NC | N | NC | NC | NC |
| A0A7J7GIN8 | NC | NC | NC | N | NC | NC | NC |
| A0A816Z962 | NC | NC | NC | N | NC | NC | NC |
| X4YI83 | NC | NC | NC | N | NC | NC | NC |
| A0A1P8AN27 | NC | NC | NC | N | NC | NC | NC |
| A0A059BEH2 | NC | NC | NC | N | NC | NC | NC |
| A0A1B5GDQ9 | NC | NC | NC | N | NC | NC | NC |
| A0A3Q7E8F1 | NC | NC | NC | N | NC | NC | NC |
| A0A6N2KT52 | NC | NC | NC | N | NC | NC | NC |
| A0A6P8CFX6 | NC | NC | NC | N | NC | NC | NC |
| A0A087HH78 | NC | NC | NC | N | NC | NC | NC |
| A0A1J6IJ09 | NC | NC | NC | N | NC | NC | NC |
| A0A1U7UVL6 | NC | NC | NC | N | NC | NC | NC |
| A0A1U8AUW6 | NC | NC | NC | N | NC | NC | NC |
| A0A5N5LS94 | NC | NC | NC | N | NC | NC | NC |
| A0A6A4PVI8 | NC | NC | NC | N | NC | NC | NC |
| A0A7N0TG95 | NC | NC | NC | N | NC | NC | NC |
| A0A843U5A2 | NC | NC | NC | N | NC | NC | NC |
| D7KYD2 | NC | NC | NC | N | NC | NC | NC |

| | | | | | | | |
|---|---|---|---|---|---|---|---|
| A0A835R4X3 | N | NC | NC | NC | NC | NC | NC |
| A0A835RJR4 | N | NC | NC | NC | NC | NC | NC |
| A0A8B7CQ09 | N | NC | NC | NC | NC | NC | NC |
| V4TLS1 | N | NC | NC | NC | NC | NC | NC |
| V4W5R9 | N | NC | NC | NC | NC | NC | NC |
| A0A6A4KSK9 | N | NC | NC | NC | NC | NC | NC |
| A0A6A5NNT0 | N | NC | NC | NC | NC | NC | NC |
| A0A2P5ABD7 | NC | NC | NC | NC | NC | NC | NC |
| A0A2P5DAK4 | NC | NC | NC | NC | NC | NC | NC |
| A0A699JR16 | NC | NC | NC | NC | NC | NC | NC |
| A0A0K9R260 | NC | NC | NC | NC | NC | NC | NC |
| A0A0R0IHB1 | NC | NC | NC | NC | NC | NC | NC |
| A0A1R3JGN3 | NC | NC | NC | NC | NC | NC | NC |
| A0A2P5BG53 | NC | NC | NC | NC | NC | NC | NC |
| A0A3P6GD58 | NC | NC | NC | NC | NC | NC | NC |
| A0A5D2WV65 | NC | NC | NC | NC | NC | NC | NC |
| A0A5N5HDA0 | NC | NC | NC | NC | NC | NC | NC |
| A0A6A3D3F6 | NC | NC | NC | NC | NC | NC | NC |
| A0A7J6EAH9 | NC | NC | NC | NC | NC | NC | NC |
| A0A7J8TW70 | NC | NC | NC | NC | NC | NC | NC |
| A0A834SRH2 | NC | NC | NC | NC | NC | NC | NC |
| D7KYD1 | NC | NC | NC | NC | NC | NC | NC |
| S8EDH9 | NC | NC | NC | NC | NC | NC | NC |
| A0A022QN30 | NC | NC | NC | NC | NC | NC | NC |
| A0A078FF12 | NC | NC | NC | NC | NC | NC | NC |
| A0A0B0MV53 | NC | NC | NC | NC | NC | NC | NC |
| A0A0D3CXF5 | NC | NC | NC | NC | NC | NC | NC |
| A0A0D3CZM7 | NC | NC | NC | NC | NC | NC | NC |
| A0A151QQ25 | NC | NC | NC | NC | NC | NC | NC |
| A0A178WGM7 | NC | NC | NC | NC | NC | NC | NC |
| A0A1J3EQ12 | NC | NC | NC | NC | NC | NC | NC |
| A0A1Q3BFA8 | NC | NC | NC | NC | NC | NC | NC |
| A0A1R3JGR1 | NC | NC | NC | NC | NC | NC | NC |
| A0A1S3YWC1 | NC | NC | NC | NC | NC | NC | NC |
| A0A1S4BJ97 | NC | NC | NC | NC | NC | NC | NC |
| A0A1U7X428 | NC | NC | NC | NC | NC | NC | NC |
| A0A200QL80 | NC | NC | NC | NC | NC | NC | NC |
| A0A251RTX1 | NC | NC | NC | NC | NC | NC | NC |
| A0A251T4I5 | NC | NC | NC | NC | NC | NC | NC |
| A0A251T5K5 | NC | NC | NC | NC | NC | NC | NC |
| A0A2C9U678 | NC | NC | NC | NC | NC | NC | NC |
| A0A2C9U6B4 | NC | NC | NC | NC | NC | NC | NC |
| A0A2C9UFE2 | NC | NC | NC | NC | NC | NC | NC |
| A0A2G9H667 | NC | NC | NC | NC | NC | NC | NC |
| A0A2I0WWC0 | NC | NC | NC | NC | NC | NC | NC |
| A0A2J6L6D8 | NC | NC | NC | NC | NC | NC | NC |
| A0A2J6L6E7 | NC | NC | NC | NC | NC | NC | NC |

| | | | | | | | |
|---|---|---|---|---|---|---|---|
| A0A2P5BG43 | NC | NC | NC | NC | NC | NC | NC |
| A0A2P5XBR6 | NC | NC | NC | NC | NC | NC | NC |
| A0A2P5YLA8 | NC | NC | NC | NC | NC | NC | NC |
| A0A2P6R3T7 | NC | NC | NC | NC | NC | NC | NC |
| A0A2R6PAT4 | NC | NC | NC | NC | NC | NC | NC |
| A0A2R6PGP4 | NC | NC | NC | NC | NC | NC | NC |
| A0A2U1NZY7 | NC | NC | NC | NC | NC | NC | NC |
| A0A2U1P4P7 | NC | NC | NC | NC | NC | NC | NC |
| A0A2U1PSW2 | NC | NC | NC | NC | NC | NC | NC |
| A0A314V176 | NC | NC | NC | NC | NC | NC | NC |
| A0A314ZJR4 | NC | NC | NC | NC | NC | NC | NC |
| A0A397YQQ5 | NC | NC | NC | NC | NC | NC | NC |
| A0A397YWD3 | NC | NC | NC | NC | NC | NC | NC |
| A0A3P6BSL4 | NC | NC | NC | NC | NC | NC | NC |
| A0A3P6DKE6 | NC | NC | NC | NC | NC | NC | NC |
| A0A445AHE6 | NC | NC | NC | NC | NC | NC | NC |
| A0A445EEM0 | NC | NC | NC | NC | NC | NC | NC |
| A0A4D9AJV2 | NC | NC | NC | NC | NC | NC | NC |
| A0A4S4E2L5 | NC | NC | NC | NC | NC | NC | NC |
| A0A540KGE2 | NC | NC | NC | NC | NC | NC | NC |
| A0A540LVM3 | NC | NC | NC | NC | NC | NC | NC |
| A0A5B6ZSC8 | NC | NC | NC | NC | NC | NC | NC |
| A0A5C7HJY8 | NC | NC | NC | NC | NC | NC | NC |
| A0A5D2EAS2 | NC | NC | NC | NC | NC | NC | NC |
| A0A5D2MXZ2 | NC | NC | NC | NC | NC | NC | NC |
| A0A5E4EFL0 | NC | NC | NC | NC | NC | NC | NC |
| A0A5J5AIQ7 | NC | NC | NC | NC | NC | NC | NC |
| A0A5J5ANC9 | NC | NC | NC | NC | NC | NC | NC |
| A0A5J5TE81 | NC | NC | NC | NC | NC | NC | NC |
| A0A5N6NRZ0 | NC | NC | NC | NC | NC | NC | NC |
| A0A5S9WR79 | NC | NC | NC | NC | NC | NC | NC |
| A0A654EMW5 | NC | NC | NC | NC | NC | NC | NC |
| A0A6A3BHK3 | NC | NC | NC | NC | NC | NC | NC |
| A0A6D2HZ60 | NC | NC | NC | NC | NC | NC | NC |
| A0A6I9SM78 | NC | NC | NC | NC | NC | NC | NC |
| A0A6J0K7P6 | NC | NC | NC | NC | NC | NC | NC |
| A0A6J0KZ28 | NC | NC | NC | NC | NC | NC | NC |
| A0A6J0L133 | NC | NC | NC | NC | NC | NC | NC |
| A0A6J0LNX2 | NC | NC | NC | NC | NC | NC | NC |
| A0A6J0M934 | NC | NC | NC | NC | NC | NC | NC |
| A0A6J1DXH5 | NC | NC | NC | NC | NC | NC | NC |
| A0A6J1FR56 | NC | NC | NC | NC | NC | NC | NC |
| A0A6J1JW16 | NC | NC | NC | NC | NC | NC | NC |
| A0A6J5TMT5 | NC | NC | NC | NC | NC | NC | NC |
| A0A6J5W2J7 | NC | NC | NC | NC | NC | NC | NC |
| A0A6P4AI34 | NC | NC | NC | NC | NC | NC | NC |
| A0A6P4PI89 | NC | NC | NC | NC | NC | NC | NC |

| | | | | | | | |
|---|---|---|---|---|---|---|---|
| A0A6P5T2V3 | NC | NC | NC | NC | NC | NC | NC |
| A0A6P5XZX1 | NC | NC | NC | NC | NC | NC | NC |
| A0A6P6V0F6 | NC | NC | NC | NC | NC | NC | NC |
| A0A6P6V9N9 | NC | NC | NC | NC | NC | NC | NC |
| A0A6S7MQ77 | NC | NC | NC | NC | NC | NC | NC |
| A0A6S7MX54 | NC | NC | NC | NC | NC | NC | NC |
| A0A7J0GZ78 | NC | NC | NC | NC | NC | NC | NC |
| A0A7J6EAQ7 | NC | NC | NC | NC | NC | NC | NC |
| A0A7J7DUZ4 | NC | NC | NC | NC | NC | NC | NC |
| A0A7J7GYA7 | NC | NC | NC | NC | NC | NC | NC |
| A0A7J8S4S9 | NC | NC | NC | NC | NC | NC | NC |
| A0A7J8V1Q0 | NC | NC | NC | NC | NC | NC | NC |
| A0A7N0TGN8 | NC | NC | NC | NC | NC | NC | NC |
| A0A7N0TGR6 | NC | NC | NC | NC | NC | NC | NC |
| A0A7N1A146 | NC | NC | NC | NC | NC | NC | NC |
| A0A7N1A8A8 | NC | NC | NC | NC | NC | NC | NC |
| A0A803M906 | NC | NC | NC | NC | NC | NC | NC |
| A0A803MFE9 | NC | NC | NC | NC | NC | NC | NC |
| A0A830BHA9 | NC | NC | NC | NC | NC | NC | NC |
| A0A834GJ13 | NC | NC | NC | NC | NC | NC | NC |
| A0A834LFQ6 | NC | NC | NC | NC | NC | NC | NC |
| A0A835JML3 | NC | NC | NC | NC | NC | NC | NC |
| A0A836DWL9 | NC | NC | NC | NC | NC | NC | NC |
| A0A836IVH9 | NC | NC | NC | NC | NC | NC | NC |
| A0A836Y2M1 | NC | NC | NC | NC | NC | NC | NC |
| A0A8B8LZA0 | NC | NC | NC | NC | NC | NC | NC |
| B9SJ16 | NC | NC | NC | NC | NC | NC | NC |
| G8A026 | NC | NC | NC | NC | NC | NC | NC |
| M4CIB5 | NC | NC | NC | NC | NC | NC | NC |
| M4DI68 | NC | NC | NC | NC | NC | NC | NC |
| M5XYV6 | NC | NC | NC | NC | NC | NC | NC |
| V7C561 | NC | NC | NC | NC | NC | NC | NC |
| A0A1U8KW11 | NC | NC | NC | NC | NC | NC | NC |
| A0A5D2MAB4 | NC | NC | NC | NC | NC | NC | NC |
| A0A5D2RUT4 | NC | NC | NC | NC | NC | NC | NC |
| A0A5D3ADX8 | NC | NC | NC | NC | NC | NC | NC |
| A0A5J5SPD5 | NC | NC | NC | NC | NC | NC | NC |
| A0A6P4NEW4 | NC | NC | NC | NC | NC | NC | NC |
| A0A7J8R1H6 | NC | NC | NC | NC | NC | NC | NC |
| A0A7J9BDB8 | NC | NC | NC | NC | NC | NC | NC |
| A0A7J9KRJ8 | NC | NC | NC | NC | NC | NC | NC |
| D7KMR2 | NC | NC | NC | NC | NC | NC | NC |
| A0A0D2PW34 | NC | NC | NC | NC | NC | NC | NC |
| A0A0D2RGN1 | NC | NC | NC | NC | NC | NC | NC |
| A0A1S3CNP4 | NC | NC | NC | NC | NC | NC | NC |
| A0A1U8NAK8 | NC | NC | NC | NC | NC | NC | NC |
| A0A1U8PQ20 | NC | NC | NC | NC | NC | NC | NC |

| | | | | | | | |
|---|---|---|---|---|---|---|---|
| A0A218W6E6 | NC | NC | NC | NC | NC | NC | NC |
| A0A2I0HX16 | NC | NC | NC | NC | NC | NC | NC |
| A0A2I4DH23 | NC | NC | NC | NC | NC | NC | NC |
| A0A2I4G1V2 | NC | NC | NC | NC | NC | NC | NC |
| A0A2K1ZGM7 | NC | NC | NC | NC | NC | NC | NC |
| A0A2P5YH81 | NC | NC | NC | NC | NC | NC | NC |
| A0A2U1KJ00 | NC | NC | NC | NC | NC | NC | NC |
| A0A438JTY9 | NC | NC | NC | NC | NC | NC | NC |
| A0A4U5M4M0 | NC | NC | NC | NC | NC | NC | NC |
| A0A4U5NL14 | NC | NC | NC | NC | NC | NC | NC |
| A0A5D2AB54 | NC | NC | NC | NC | NC | NC | NC |
| A0A5D2EA32 | NC | NC | NC | NC | NC | NC | NC |
| A0A5D2HM80 | NC | NC | NC | NC | NC | NC | NC |
| A0A5D2IAT6 | NC | NC | NC | NC | NC | NC | NC |
| A0A5D2MZF2 | NC | NC | NC | NC | NC | NC | NC |
| A0A5D2RSQ7 | NC | NC | NC | NC | NC | NC | NC |
| A0A5J5NZR4 | NC | NC | NC | NC | NC | NC | NC |
| A0A5J5TBC0 | NC | NC | NC | NC | NC | NC | NC |
| A0A6A1UZI7 | NC | NC | NC | NC | NC | NC | NC |
| A0A6A2Z699 | NC | NC | NC | NC | NC | NC | NC |
| A0A6J1B1S2 | NC | NC | NC | NC | NC | NC | NC |
| A0A6P6BF05 | NC | NC | NC | NC | NC | NC | NC |
| A0A6P8CE95 | NC | NC | NC | NC | NC | NC | NC |
| A0A7J8MEX9 | NC | NC | NC | NC | NC | NC | NC |
| A0A7J9KGT5 | NC | NC | NC | NC | NC | NC | NC |
| A0A7N2L689 | NC | NC | NC | NC | NC | NC | NC |
| A0A833WSM5 | NC | NC | NC | NC | NC | NC | NC |
| A0A833WXW0 | NC | NC | NC | NC | NC | NC | NC |
| A0A8B8P304 | NC | NC | NC | NC | NC | NC | NC |
| D7T8M1 | NC | NC | NC | NC | NC | NC | NC |
| R0GL28 | NC | NC | NC | NC | NC | NC | NC |
| A0A6P5XDT9 | NC | NC | NC | NC | NC | NC | NC |
| A0A067L0U1 | NC | NC | NC | NC | NC | NC | NC |
| A0A078FBY4 | NC | NC | NC | NC | NC | NC | NC |
| A0A0D3APP7 | NC | NC | NC | NC | NC | NC | NC |
| A0A124SDQ4 | NC | NC | NC | NC | NC | NC | NC |
| A0A251RU16 | NC | NC | NC | NC | NC | NC | NC |
| A0A251T5P0 | NC | NC | NC | NC | NC | NC | NC |
| A0A2G2ZHF1 | NC | NC | NC | NC | NC | NC | NC |
| A0A2G3CFB2 | NC | NC | NC | NC | NC | NC | NC |
| A0A2P5BG63 | NC | NC | NC | NC | NC | NC | NC |
| A0A2U1KIZ8 | NC | NC | NC | NC | NC | NC | NC |
| A0A398AAR3 | NC | NC | NC | NC | NC | NC | NC |
| A0A3N6R5G3 | NC | NC | NC | NC | NC | NC | NC |
| A0A3N7H945 | NC | NC | NC | NC | NC | NC | NC |
| A0A3P6ASC2 | NC | NC | NC | NC | NC | NC | NC |
| A0A5N6PVF8 | NC | NC | NC | NC | NC | NC | NC |

| | | | | | | | |
|---|---|---|---|---|---|---|---|
| A0A6A3B0V7 | NC | NC | NC | NC | NC | NC | NC |
| A0A6A5LU17 | NC | NC | NC | NC | NC | NC | NC |
| A0A6J1FMQ8 | NC | NC | NC | NC | NC | NC | NC |
| A0A6J1ICU8 | NC | NC | NC | NC | NC | NC | NC |
| A0A6L2JQZ0 | NC | NC | NC | NC | NC | NC | NC |
| A0A7J0DR01 | NC | NC | NC | NC | NC | NC | NC |
| A0A7J6V4C1 | NC | NC | NC | NC | NC | NC | NC |
| A0A8J5YDT0 | NC | NC | NC | NC | NC | NC | NC |
| M1CVR0 | NC | NC | NC | NC | NC | NC | NC |
| M4CUN9 | NC | NC | NC | NC | NC | NC | NC |
| S8DZ20 | NC | NC | NC | NC | NC | NC | NC |
| A0A5D3D9U0 | NC | NC | NC | NC | NC | NC | NC |
| R0GCI8 | NC | NC | NC | NC | NC | NC | NC |
| B3H7A9 | NC | NC | NC | NC | NC | NC | NC |
| A0A8D9H5V4 | NC | NC | NC | NC | NC | NC | NC |
| A0A0D2QXV4 | NC | NC | NC | NC | NC | NC | NC |
| A0A1U8KVZ6 | NC | NC | NC | NC | NC | NC | NC |
| A0A5D2DR09 | NC | NC | NC | NC | NC | NC | NC |
| A0A5D2MAK8 | NC | NC | NC | NC | NC | NC | NC |
| A0A5J5SVA8 | NC | NC | NC | NC | NC | NC | NC |
| A0A0B0N5Y7 | NC | NC | NC | NC | NC | NC | NC |
| A0A5D3AJX5 | NC | NC | NC | NC | NC | NC | NC |
| A0A6M2F086 | NC | NC | NC | NC | NC | NC | NC |
| A0A816KI15 | NC | NC | NC | NC | NC | NC | NC |
| A0A3Q7X6X3 | NCN | NC | NC | NC | NC | NC | NC |
| A0A8J5CAK2 | NCN | NC | NC | NCN | NC | NC | NC |
| A0A2H5PVZ2 | NCN | NC | NC | NCN | NC | NC | NC |
| A0A1D6JA94 | CNC | NC | NC | CNC | NCN | NC | NC |
| A0A498HFS9 | NCN | NC | NC | CNC | NCN | NC | NC |
| A0A0D3GEZ0 | N | NC | NC | N | NCN | NC | NC |
| V4MRQ6 | N | NC | NC | N | NCN | NC | NC |
| A0A0E0LA27 | N | NC | NC | N | NCN | NC | NC |
| A0A2K1KE32 | N | NC | NC | N | CNC | NCN | NC |
| A0A484MHA3 | NC | NC | NC | CN | NC | NCN | NC |
| A0A4Y7JL46 | N | NC | NC | N | NC | NCN | NC |
| A0A2P6VHX5 | N | N | NCN | N | NC | NCN | NC |
| A0A5D2SH05 | NCN | NC | NC | NCN | NC | NCN | NC |
| A0A7J6WA12 | CNC | CN | NCN | CNC | CN | CN | NCN |
| A0A835UF66 | N | CN | NCN | CN | CN | CNC | NCN |
| A0A835UBZ1 | N | CN | NCN | CN | CN | CNC | NCN |
| A0A2U1Q6M5 | NCN | CN | NCN | CN | CN | CNC | NCN |
| A0A103Y0S4 | NCN | CN | NCN | CN | CN | CNC | NCN |
| A0A6A5KZZ5 | NC | CN | NCN | CN | CNC | CNC | NCN |
| A0A6A4LAQ9 | CN | CNC | NCN | CNC | CNC | CNC | NCN |
| A0A0E0K4Z3 | NCN | NC | CNC | CNC | NC | CNC | NCN |
| A0A1R3GRG1 | NC | NCN | NCN | NCN | NC | CNC | NCN |
| A0A803M907 | NCN | NCN | NCN | NCN | NC | CNC | NCN |

| | | | | | | | |
|---|---|---|---|---|---|---|---|
| A0A4S4DRW9 | N | NCN | NCN | CNC | NC | NC | NCN |
| A0A5D2SEX9 | NCN | NCN | NCN | NCN | NCN | NCN | NCN |
| A0A061E7V0 | NCN | NCN | NCN | NCN | NCN | NCN | NCN |
